# Persistent Neanderthal occupation of the open-air site of ‘Ein Qashish, Israel

**DOI:** 10.1371/journal.pone.0215668

**Published:** 2019-06-26

**Authors:** Ravid Ekshtain, Ariel Malinsky-Buller, Noam Greenbaum, Netta Mitki, Mareike C. Stahlschmidt, Ruth Shahack-Gross, Nadav Nir, Naomi Porat, Daniella E. Bar-Yosef Mayer, Reuven Yeshurun, Ella Been, Yoel Rak, Nuha Agha, Lena Brailovsky, Masha Krakovsky, Polina Spivak, Micka Ullman, Ariel Vered, Omry Barzilai, Erella Hovers

**Affiliations:** 1 The Institute of Archaeology, the Hebrew University of Jerusalem, Jerusalem, Israel; 2 MONREPOS Archaeological Research Centre and Museum for Human Behavioural Evolution, Germany; 3 Department of Geography & Environmental Studies, University of Haifa, Haifa, Israel; 4 School of Archaeology, University College Dublin, Dublin, Ireland; 5 Department of Maritime Civilizations, Recanati Institute of Maritime Studies, Charney School of Marine Sciences, University of Haifa, Haifa, Israel; 6 Geological Survey of Israel, Jerusalem, Israel; 7 The Steinhardt Museum of Natural History, Tel Aviv University, Tel Aviv, Israel; 8 Zinman Institute of Archaeology, University of Haifa, Haifa, Israel; 9 Faculty of Health Professions, Ono Academic College, Kiryat Ono, Israel; 10 Department of Anatomy and Anthropology, Sackler Faculty of Medicine, Tel Aviv University, Tel Aviv, Israel; 11 Israel Antiquities Authority, Jerusalem, Israel; 12 Department of Bible, Archaeology, and Ancient Near Eastern Studies, Ben-Gurion University of the Negev, Beer-Sheva, Israel; Max Planck Institute for the Science of Human History, GERMANY

## Abstract

Over the last two decades, much of the recent efforts dedicated to the Levantine Middle Paleolithic has concentrated on the role of open-air sites in the settlement system in the region. Here focus on the site of ‘Ein Qashish as a cases study. Located in present-day northern Israel, the area of this site is estimated to have been >1300 m^2^, of which ca. 670 were excavated. The site is located at the confluence of the Qishon stream with a small tributary running off the eastern flanks of the Mt. Carmel. At the area of this confluence, water channels and alluvial deposits created a dynamic depositional environment. Four Archaeological Units were identified in a 4.5-m thick stratigraphic sequence were dated by Optically Stimulated Luminescence (OSL) to between—71 and 54 ka, and probably shorter time span–~70-~60 ka. Here we present the diverse material culture remains from the site (lithics, including refitted sequences; modified limestone pieces; molluscs; faunal remains) against their changing paleogeographic backdrop. Skeletal evidence suggests that these remains were associated with Neanderthals. The large-scale repeated accumulation of late Middle Paleolithic remains in the same place on the landscape provides a unique opportunity to address questions of occupation duration and intensity in open-air sites. We find that each occupation was of ephemeral nature, yet presents a range of activities, suggesting that the locale has been used as a generalized residential site rather than specialized task-specific ones. This role of ‘Ein Qashish did not change through time, suggesting that during the late Middle Paleolithic settlement system in this part of the southern Levant were stable.

## Introduction

The location of sites on the ancient landscape is the result of complex decision-making that was based on the environmental, ecological and social preferences of prehistoric groups. Researchers have approached the issue of location decisions from a dichotomous perspective placing sheltered (caves, rock shelters) habitation sites vs. short-term task specific open-air sites. Such an approach is anchored in the perception biases of both prehistoric groups and present-day scholars. In the past, the fixed locations of sheltered sites and their visibility on the paleo-landscape drew the attention of humans, leading to repeated occupations that formed long and rich sequences. Although closed sites provide less opportunities for resource procurement (e.g., they cannot be used for hunting or for raw material acquisition), they offer better shelter especially to the more vulnerable members of the group (very young; very old; pregnant females), and are likely to preserve the variable (albeit time-averaged) signatures of social groups. Combined with their visibility on the modern landscape, such characteristics have attracted prehistorians, who targeted sheltered sites as their primary research focus and were often rewarded by spectacular findings. This in turn often biased research in favor of sheltered sites (see [1,2]: appendix 3 for the Levant and [3] for the European record). In the Levant, specifically, material culture remains from caves (mainly stone tools and bones) have constituted the main source of information for comprehending Middle Paleolithic lifeways. In contrast, the location of open-air would often be decided upon in relation to specific traits or activities (e.g., permanent water sources, raw material procurement, hunting and/or plant processing) that cannot take place within sheltered sites. The range of behavioral activities at open-air sites may be more variable than that of cave sites [4,5]. Still, the lack of physical boundaries to such sites suggested to many researchers that prehistoric groups did not necessarily return to the exact same spot on the landscape, rendering excavated sequences shorter and less useful for diachronic studies. Also, open-air sites are susceptible to landscape-scale processes in addition to localized anthropogenic, geochemical and taphonomic depositional processes (e.g., [1,6–10]), which affect both site preservation and opportunities for archaeological discovery.

While Levantine Middle Paleolithic (MP) open-air sites were studied in the past [11–18], a renewed research over the last two decades has focused on this type of sites as a source of information complementary to that provided by sheltered sites (e.g.,[8,19–29]). This has resulted in new perspectives on land-use and mobility patterns of Levantine MP hunter-gatherers.

**Fig 1:**
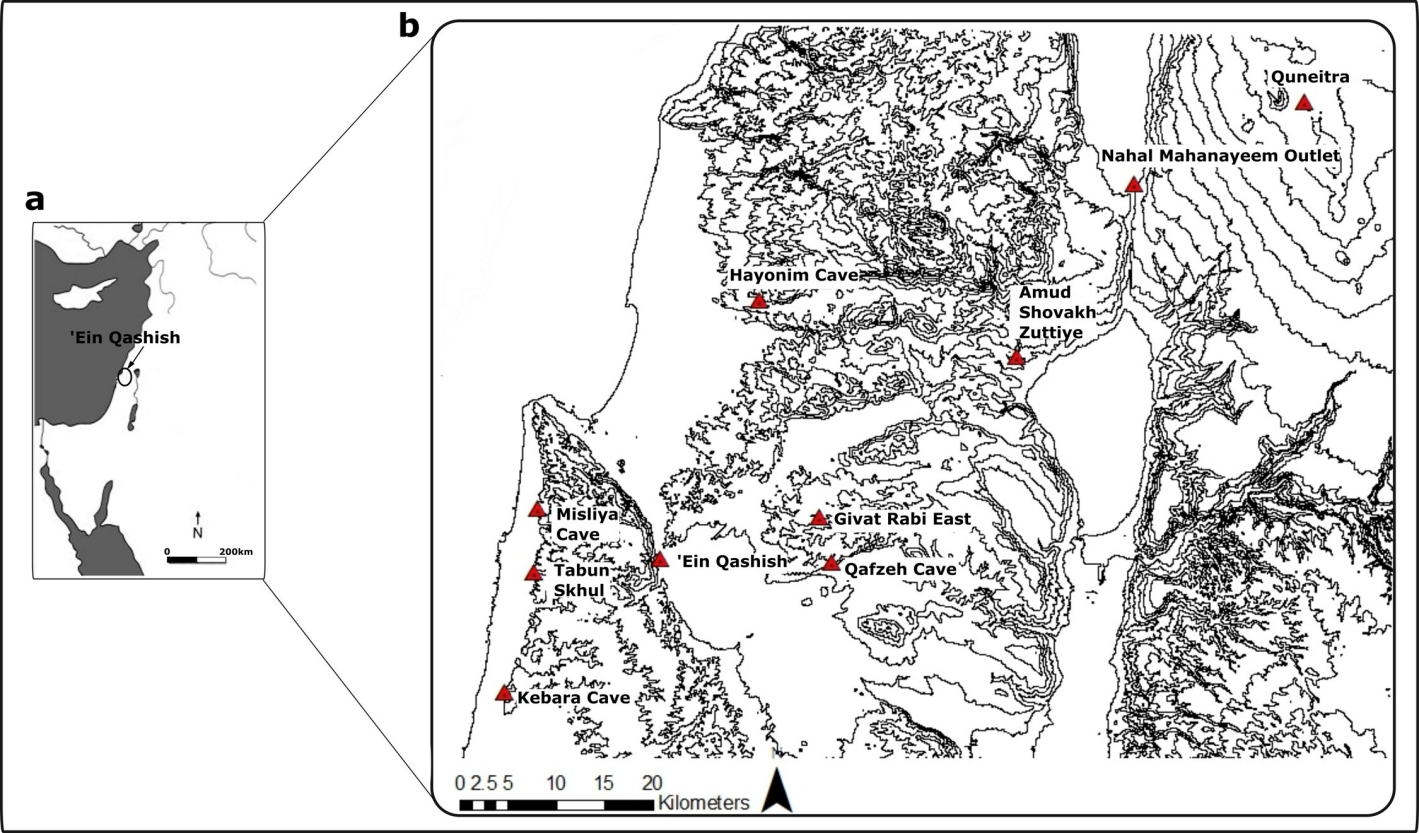
MP sites in northern Israel. Map: drown by R. Ekshtain using ArcMap 10.6.

**Fig 2:**
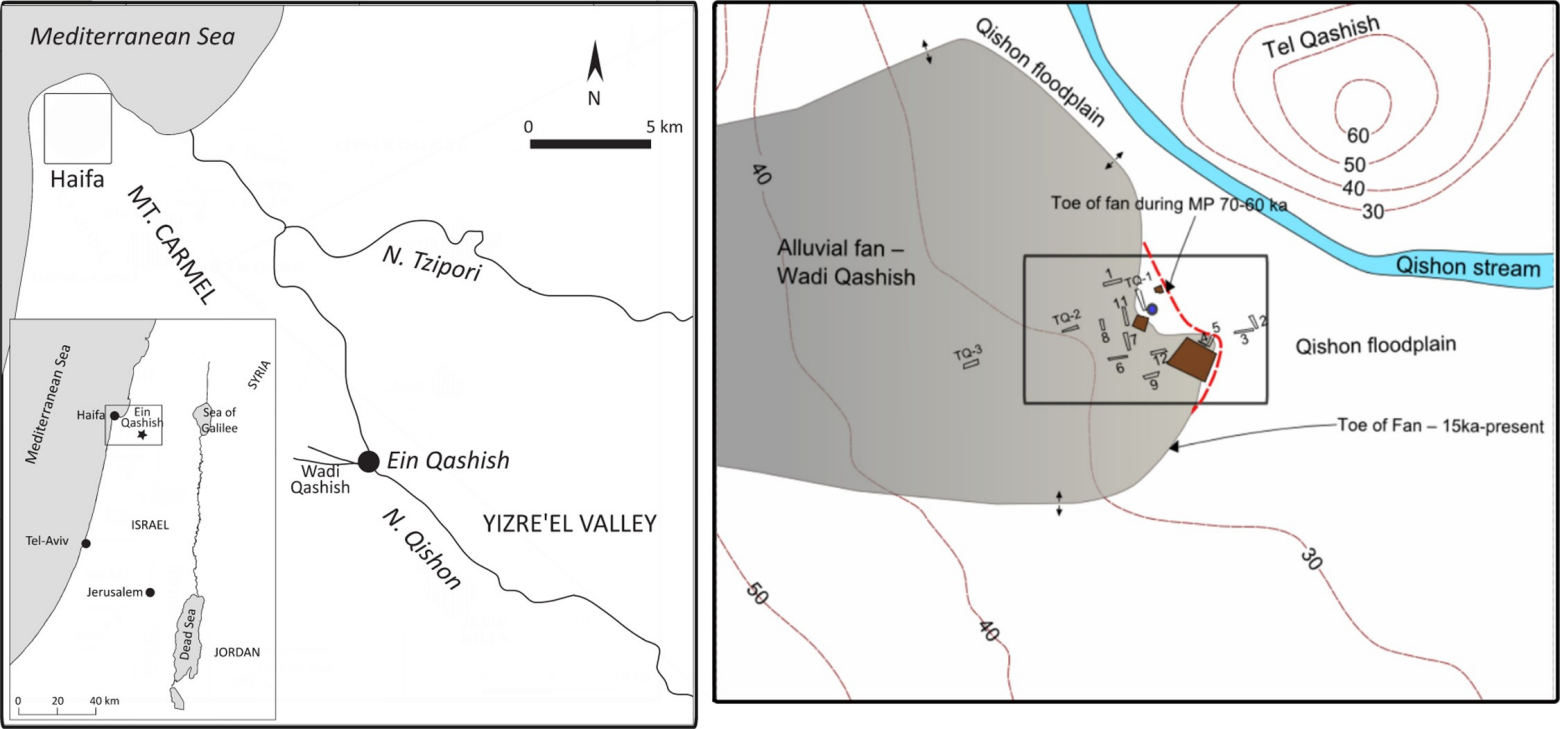
Location of the site of ‘Ein Qashish. Left: The location of ‘Ein Qashish in the Yizra’el valley. Right: a close-up map of the site’s environments. Grey area represents the present-day alluvial fan of Wadi Qashish. The changes in the fan’s eastern boundary during the occupations of the Middle Paleolithic (red dashed line). Excavation areas are shown as brown rectangles. TQ-1 –TQ-3 are geological trenches excavated during 2009–2011. Otherwise, trench numbers represent geological trenches made by IAA in 2012. Map down by R. Ekshtain using ArcMap 10.6.

**Fig 3:**
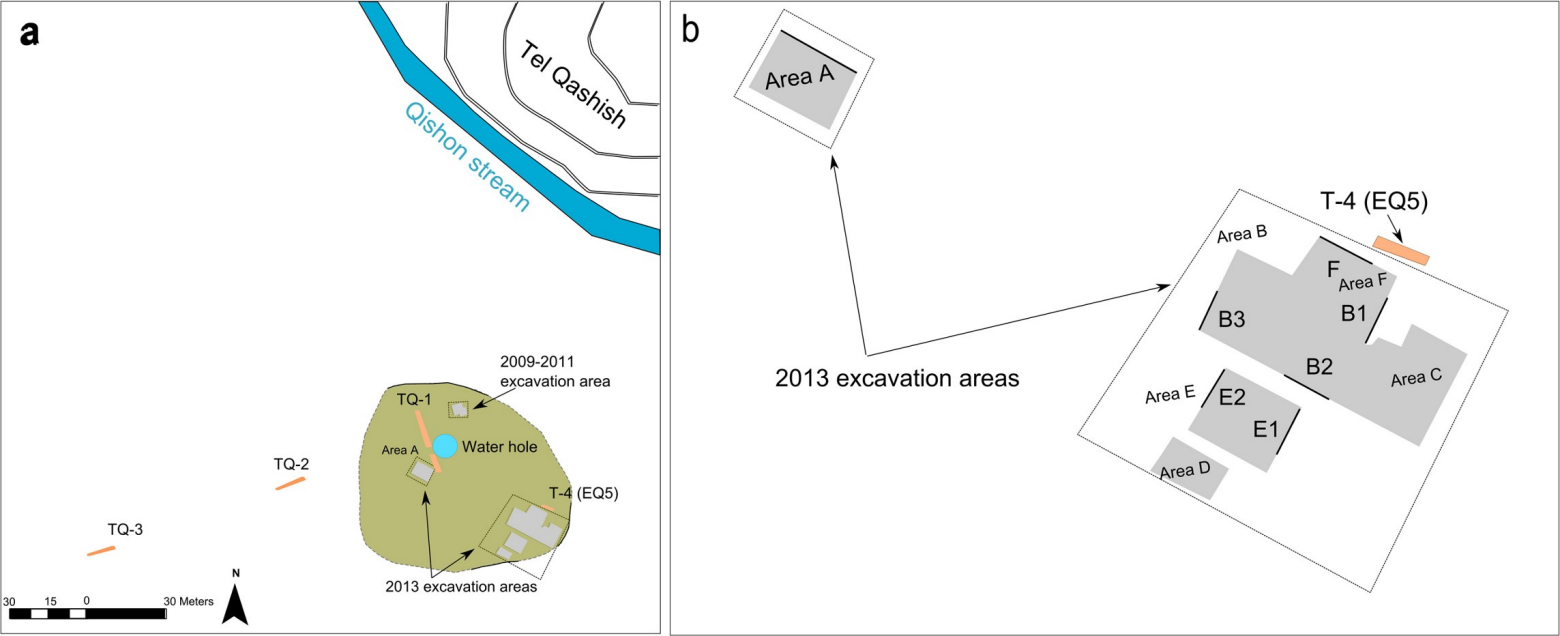
The ‘Ein Qashish excavation areas. Left: Spatial relationship between the 2009–2011 and 2013 excavation areas. The green area represents the extent of the archaeological site as reconstructed from the excavated areas and geological trenches. Right: Map of the 2013 excavation areas. Black lines represent indicative sections shown in Fig 4. Map down by R. Ekshtain using ArcMap 10.6.

‘Ein Qashish (EQ) is a MP open-air site located in northern Israel in the northwestern part of the Yizra’el Valley on the bank of the Qishon stream. It is situated in proximity to a number of MP open-air and cave sites (Fig 1). First discovered in 2004, the site is known from three series of excavations (in 2005, 2009–2011 and 2013) as well as geological trenching [8,19,30–33]. The 2013 season, the focus of the current study, was conducted as a salvage excavation preceding major road construction. A total extent of ~ 670 m² was excavated in several areas, including a number of stratigraphic profiles (Figs 2 and 3). This renders it one of the largest excavations of MP sites in the Levant. The site’s lateral extent, as reconstructed from the occurrence of MP archaeological material in the excavations and in nearby geological trenches, is estimated to have been >1300 m^2^. Four archaeological units were recognized, located at depths between 3.5–4.5 meters below the present-day surface, in the various areas of excavation. A series of Optically Stimulated Luminescence (OSL) ages places all the units within a maximal time-span of 17 kyr, between—71 and 54 ka, and probably a shorter time span of ~70-~60 ka [8,19,33].

This paper explores proxies of the activities that took place at the site and how activities as well as occupation intensity may have changed diachronically. The unusual large scale of the excavations at the site, as well as the sequential accumulation of archaeological material at the same locale, present a unique opportunity to explore the role of an open-air locality within hominin settlement systems in a dynamic micro-habitat.

We present the geological and cultural stratigraphy of the site, taking into account the effects of various depositional processes [8,34]. Material culture remains are then presented in their contexts within the four main MP occupation units.

Our results suggest that EQ was a focal point of a variety of activities. The site was located in an attractive ecological setting and therefore occupied repeatedly during the MP. Through time, this locality may have played a permanent role in the settlement dynamics of the Levantine late MP groups in northern Israel.

## Materials and methods

The 2013 excavations at the site were conducted under permit # A-6686 from the Israel Antiquity Authority as a salvage excavation after accidental damage to the then-known MP deposits. The study of finds from this excavation has been conducted under the same permit.

The excavation was carried out in six Areas (A–F), totaling ~670 m2. All the areas were excavated according to a single grid system and aligned to the Israel Grid System coordinates. All the excavated sediments were dry-sieved; ca. 15% were sampled by wet sieving.

The stratigraphy of the EQ area was studied from stratigraphic trenches and the excavation areas. Representative profiles were examined in detail and described for their stratigraphy and for the properties of the sedimentary units. The major units were sampled for OSL dating and for chemical and mineralogical analyses. The stratigraphy of each trench was established based on the field relations between the various units, the results of OSL dating and the properties of the sedimentary units including the presence of archaeological artifacts. Bulk composition, clay mineralogy using XRD and OSL dating were carried out in the laboratories of the Geological Survey of Israel (GSI). Micromorphological work was conducted at the Kimmel Center, the Weizmann Institute of Science. The results of latter work have been reported in [34].

After the site’ stratigraphic sequence was established, samples for OSL dating were collected from freshly cleaned profiles in the different excavation areas. In order to prevent exposure to sunlight, sampling took place underneath dark hoods and the samples were immediately in black, light-proof bags. Several samples were collected from each stratigraphic unit, and a complementary sample for dose rate measurements was taken from each sampling location. Detailed laboratory methods are published in [33], and are applicable to the larger data set reported in this paper.

Artifacts larger than 20 mm were mapped three-dimensionally using Total Station instruments (Sokkia 630 and Leica FTD 05), and each item was given a unique ID number according to a number bank. Each unique number was preceded by the letters identifying the material (“F” for lithic items, “B” for bones, “H” for hominin bones, “A” for tentative anvil [see below] and “M” for mollusks). Artifacts that could not be mapped were collected and bagged by excavation squares of 1 m^2^ and 5 cm vertical spit each. These items were later inventoried in the laboratory, using the same number bank. Site maps and distribution maps of various finds were created using ArcMap 10.6.

Items are stored at the Institute of Archaeology, The Hebrew University of Jerusalem. The hominin remains are on loan to the department of anatomy, the Medical School of Tel Aviv University. All items are accessible by request until final publication, at which time they will become publicly available.

The lithic items undergo a detailed attribute analysis based on the variables and attributes used in the analyses of Levantine Middle Paleolithic lithic assemblages (see [20] and references therein). Faunal analyses focused on taxonomy, anatomical breakdown and taphonomic signature, as reported (on a different sample) by [8].

## Upper pleistocene and holocene stratigraphy and depositional environments in the area of ‘Ein Qashish

The stratigraphy of the site is influenced by its location at the contact zone between the gravel-and-reddish clay alluvial fan of Wadi Qashish and the silty clay floodplain of the Qishon stream. The sediments that contain the archaeological materials generally consist of black to greyish-brown loamy clay deposited along the Qishon stream by low-energy flows and accumulated as floodplain/overbank deposits. Throughout the sequence the sediments are dominated by smectitic clay derived from thick vertisols upstream. In addition, the sediments contain aeolian silt and windblown marine fine sand, and are rich in aeolian and reworked quartz and calcite [19,33,35]. Gravel clusters, present in some of the units, may be related to rare larger floods of the Qishon stream. It is more likely, however, that such gravels derived from episodic flows from a steep tributary, Wadi Qashish, which runs off the eastern flanks of Mt. Carmel (Fig 2), indicating the toe of its alluvial fan. The field relations between the clay and gravel differ spatially from one place to another on the floodplain and diachronically, as is apparent through the sequence within each excavation area. The shifts in the location of the dynamic contacts between the two facies are controlled by the fluvial activity of the ephemeral Wadi Qashish and the perennial flows in the Qishon stream. The increased fluvial activity of Wadi Qashish between 15–10 ka pushed the alluvial fan contact eastward into the Qishon floodplain and changed its location compared to its presumed location during MP times (Fig 2 and see below).

**Fig 4:**
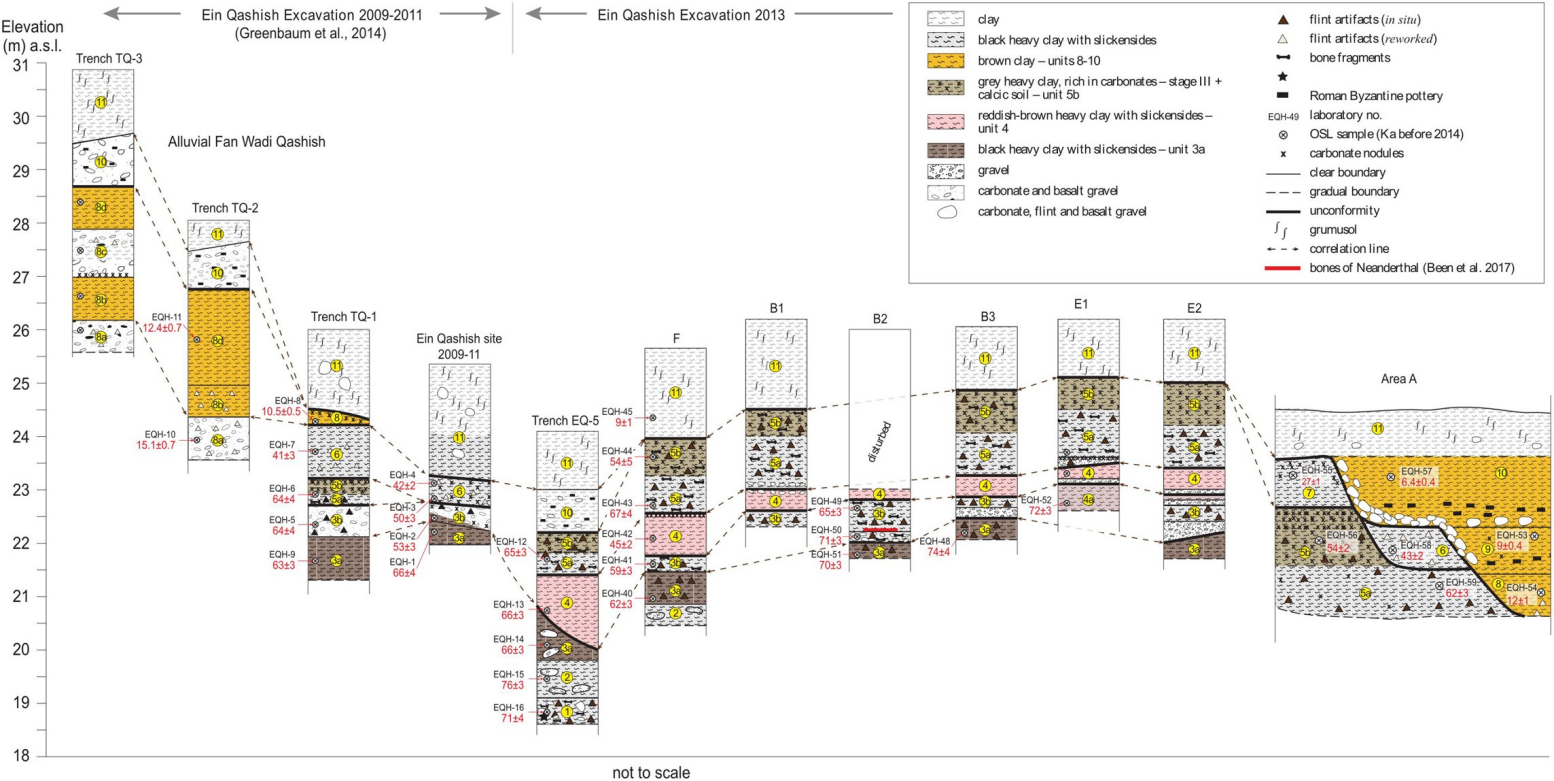
Stratigraphic correlations and OSL chronology across the ‘Ein Qashish site. Drawing: N. Yoselevich.

The stratigraphic scheme used here is based on correlations across all the excavation areas and trenches exposed during the various seasons of fieldwork (Table 1; and S1 section) and revised for the purpose of this study on the basis of new dating, sedimentological and mineralogical data. Following this revision, we now use the terminology of ‘units’ instead of ‘layers’ (previously used by [32] and [33]. Units are numbered (1–11) from the oldest to youngest (see S1 section and S1 Table for details). The field relations and stratigraphic integration among all excavation areas and trenches are presented in Figs 3 and 4 and in Table 1.

**Table 1:** Description of sedimentological units and stratigraphic correlations across the site of ‘Ein Qashish and OSL dates (see also Fig 4 and S1 section and S1 Table for details).

Unit	2009–2011 excavation & trenches [19]	Presence of MP archaeology	Description	Depositional context	OSL ages [19](ka)	OSL 2013 excavation [33](ka)	OSL ages area A(ka)	Average OSL ages(ka)
1		present	black loamy clay+ coarse gravel	*Phase I*: Alluvial		EQ-5 71±4		71±4 (N = 1)
2			black loamy clay+ lenses of small pebbles	*Phase I*: Alluvial		EQ-5 73±3		76±3 (N = 1)
3a	1	present	black loamy clay+ hydromorphic, reduction features, gypsum crystals	*Phase II*: Shallow marsh	EQ2009-11 66±4 TQ-1 63±3	EQ-5 66±3, Sec. F 62±3, Sec. B2 70±3, Sec. B3 74±4		68±5 (N = 6)
3b	2	present	limestone, flint dolomite, chalk gravel+ black loamy clay matrix	*Phase II*: Shallow marsh	EQ 2009–11 53±3 TQ-1 64±4	Sec. F 59±3, Sec. B2 71±3, Sec. B2 65±3		65±6 (N = 5)
4			reddish-brown loamy clay	*Phase III*: Seasonalwater body		EQ5 66± 3, Sec. F 45± 2		56±15 (N = 4)
5a	3	present	black-grey loamy clay+ hydromorphic, reduction features	*Phase IV*: Shallow marsh	TQ-1 64±4	EQ-5 65±3, Sec. F 67±4	62±3	66±2 (N = 5)
5b	3	present	grey loamy clay+ well developed calcic soil	*Phase IV*: Shallow marsh, drying, pedogenesis		Sec. F 54±5	54±2	54±5 (N = 3)
6	4		black loamy clay+ carbonate nodules–initial calcic soil	*Phase V*: Shallow marsh, drying pedogenesis	EQ 2009–11 50±3, EQ 2009–11, 42±2, TQ-1 41±3		43±2	44±3 (N = 4)
7			black loamy clay+ carbonate nodules–initial calcic soil	*Phase VI*: Shallow marsh, drying, pedogenesis			27±1	27±1 (N = 1)
8	5, 6a, 6b,6c		gravel+ brown loamy clay from Wadi Qashish	*Phase VII*: Late Pleistocene, Alluvial, high-energy	TQ-1 10.5±0.5, TQ-2 12.4±0.7, TQ-3 15.1±0.7		12±1	12.5±0.7 (N = 4)
9	7		brown loamy clay Chalcolithic ceramics	*Phase VIII*: Holocene, Alluvial, low-energy and stabilization			9±0.4	9±0.4 (N = 1)
10	8		gravel+ loamy clay+ Chalcolithic, Roman, Byzantine ceramics	*Phase VIII*: Holocene, Alluvial, low-energy and stabilization			6.4±0.4	6.4±0.4 (N = 1)
11	9		grumusol	*Phase VIII*: Holocene stabilization		<9±1		<9±1 (N = 1)

The general stratigraphy at the site reflects the geological history of the Qishon stream during approximately the last 70 kyr. The continuous deposition and aggradation of the fine alluvial overbank sediments, which originate from the upper part of the basin, is interrupted by several unconformities. Some of these unconformities probably correspond to short periods, such as between Unit 3b and Unit 4 and between Unit 4 and Unit 5a. The OSL dating could not distinguish clearly between the ages of the lowermost and uppermost MP layers in units 1–5, and it appears that the sediments were deposited rapidly (Tables 1 and S1 and supplementary material in [33]. Given the resolution of the OSL dates and the dynamic processes affecting the area, a tentative average rate of deposition is three centimeters in one thousand years.

The site presents eight main depositional phases. The first phase (*phase I*, Table 1*)*, represented by Units 1 and 2, is alluvial. The sediments consist of coarse and fine gravel lens within a silty clay matrix, indicating a fluvially- dynamic environment, without indications of marsh development or reducing conditions, nor pedogenic properties. The second phase (depositional *phase II*) is an inland, shallow marsh environment [36,37], developed over the Qishon floodplain and represented by Unit 3 (sub-units 3a and 3b). During the third phase (depositional *phase III)* a limited seasonal water body was formed (Unit 4). A second marsh environment developed during depositional *phase IV* which is represented by Unit 5 (sub-units 5a-5b).

The characteristics of the various marshes differ. During the two marsh *phases II* and *IV*, sedimentary units include features that indicate hydromorphic conditions and water saturation, such as gley, mottling, Fe-Mn concretions and stains, increasing with depth and proximity to the local modern-day groundwater level. The presence of rhizoliths indicates that these sediments served as substrate for vegetation, the surface of the marsh was stabilized and exposed to aerobic processes and oxidation. This suggests that the marsh may had been seasonal or discontinuous. These observations are consistent with the results of macromorphological [19] and micromorphological [35] studies of sediments from Units 1–3 in the 2009 excavations north of the site (i.e., equivalent to Units 3 and 5 of the 2013 excavations). On the basis of micromorphological and geochemical studies, it was suggested that *phase III* (Unit 4) was a water body extending across an area of at least 600 m^2^ [34,38]. The Fe-rich, reddish clay and the presence of large gypsum crystals indicate cycles of dissolution and re-precipitation through evaporation from the proposed shallow water body. This suggests the occurrence of short-term reducing conditions followed by vegetation cover/oxidation and evaporation, consistent with a seasonal rather than permanent water body. The presence of slickensides, formed by shrink-swell cycles, testifies to wetting and drying episodes as well as argilliturbation.

The well-developed calcic soil (S2 section) overlying the marsh deposit of Unit 5b developed on an exposed and stable surface over a time span <11 kyr. The site’s area was then covered by a third marsh phase (*Phase V*) at about 43 ka (Unit 6), with macro-properties similar to those of the earlier marshes. This was followed by a fourth marsh (*Phase VI*) at about 27 ka (Unit 7). A parallel marsh episode around 30 ka was documented by [39] in cores from Haifa Bay. An unconformity between *phases V* and *VI* does not allow us to reconstruct the environment between 43 and 27 ka.

The formation of the major marsh phases was related to blockage of the narrow Qishon water gap by sand transported by westerly winds during periods of low sea level. At such times the coastline would have been several km to the west, the continental shelf exposed [19,36,40–42], and the climate was wetter. This suggestion is supported by the traces of marine sand at the site [35] as well as by adsorption of barium and chlorine, elements that are associated with seawater spray, in Unit 4 [34,38].

Large floods in the Qishon stream, which opened the sand blockages and drained the marshes, were related to Heinrich events [19,43]. The accumulation of Unit 7 (*Phase VI*) was followed by a <11 kyr long period of exposure and pedogenesis, as indicated by the presence of a moderately-developed calcic soil at the top of the unit. The alternating gravelly and reddish-brown clay sedimentary complex of Unit 8 (*phase VII*) is interpreted as stemming from large, energetic flows from Wadi Qashish into the alluvial fan in the then-deserted area of the MP site, at 15 to 10 ka. In some places, the flows along the alluvial fan cut the MP landscape, depositing large gravel in channels, whereas in other places the area was covered with reddish-brown clay. This period of exposure is also documented at the nearby Epi-Paleolithic site of Ein Qashish South [44]. This site, which is located a few hundred meters south of the MP site and further away from the Qishon stream, was occupied by Kebaran, Geometric Kebaran and Natufian settlements (Units 6, 5, 4 in Fig 3 within [44]), radiocarbon and OSL dated between 25 and 14 ka. The time span of 25–14 ka, corresponding to the LGM and post-LGM periods, was probably characterized by large floods along the Qishon stream (Fig 9 within [19]). The 0.5 m-thick sedimentary section deposited during this period at ‘Ein Qashish South which is probably partly anthropogenic, includes also large angular rock clasts, clay and some sand, suggesting that this site was away from the Qishon floodplain and closer to the slopes of Mt. Carmel. The sediments that accumulated during the Natufian time at ‘Ein Qashish South were truncated at a similar time as the truncation and coverage of the MP site by Unit 8 (*phase VII*). Stabilization and soil development characterize the end of this period as shown in trench TQ-1 (Fig 4).

The Holocene deposits (Unit 9 to Unit 11, *phase VIII*) are composed of dark brown clay with some gravel and contain pottery of various periods, as well as black clays deposited from time to time during larger floods along the Qishon stream.

MP human activities were exposed in four main depositional units: 3a, 3b, 5a and 5b, spanning a maximum time period to ~ 71–54 ka. Unit 1, in which there were MP lithics, was only exposed in geological trenching but not in the excavation areas, and therefore will not be discussed further. A micromorphological study suggests that fluvial and argilliturbation reworking of sediments in the different areas and various stratigraphic units was limited to the scale of a few centimeters to a few decimeters [34]. Because of the high variability of syn- and post-depositional processes at these vertical and lateral scales, some areas at the site present pristine configurations of artifacts and/or faunal remains [8], see below), whereas disturbances in other parts (e.g., 2009–2011 area) of the site are constrained by the scale of the fluvial and turbation process [31,34].

## The archaeological sequence

The rich archaeological record of EQ consists of lithics, faunal remains and a few unusual finds such as antlers, a marine mollusk, potentially anvils and human remains.

**Fig 5:**
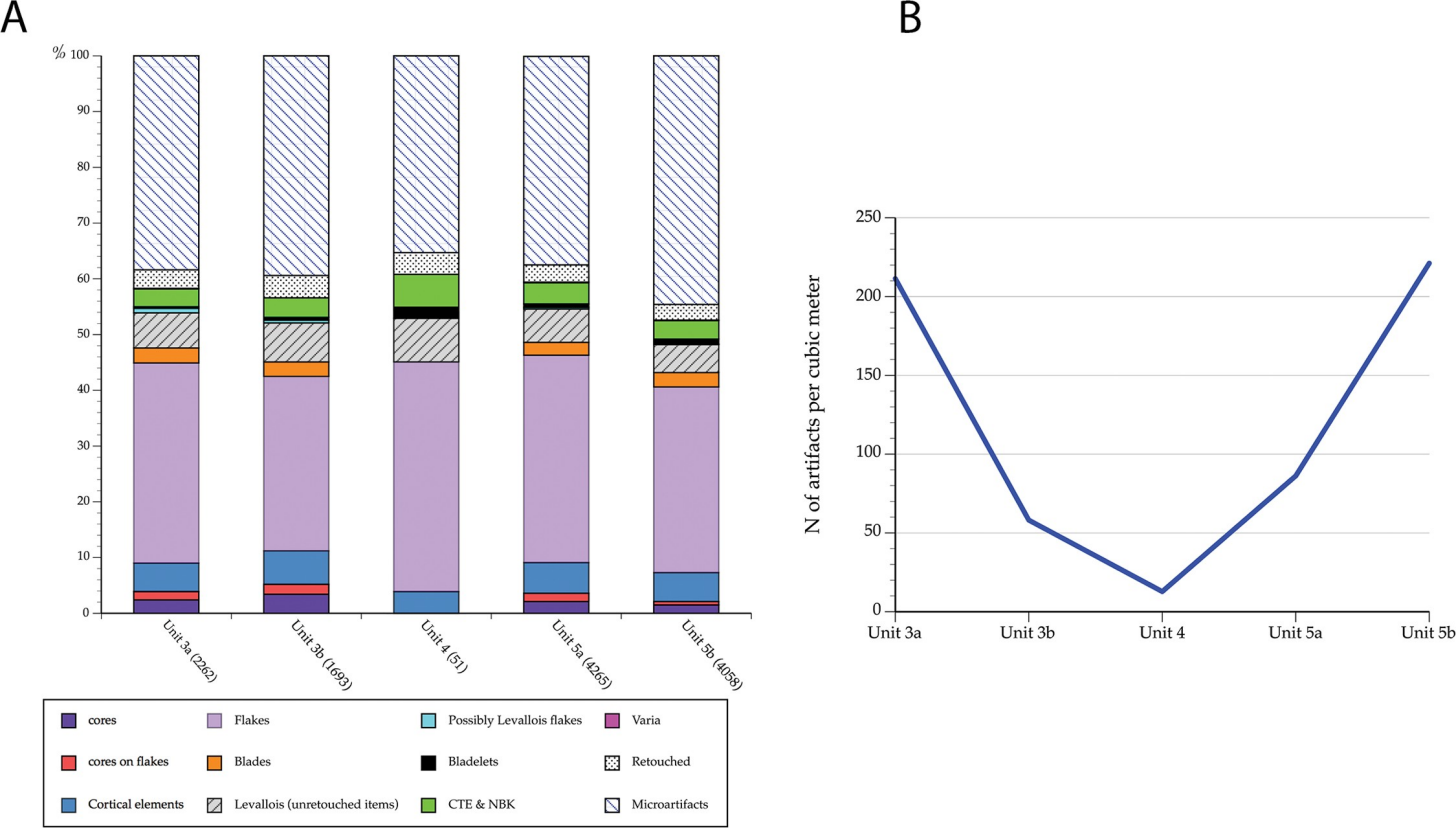
Assemblage composition and artifact densities. A. Frequencies of artifacts by lithic technological categories in the archaeological units at EQ. B. Overall artifact densities (n/m^3^) along the stratigraphic sequence. Note that we present corrected data (see text) for Units 5a and 5b. Raw data for this figure are provided in Table b in S2 Table and in S3 Table.

There are 12,329 lithic artifacts from all the 2013 excavated areas. Of these, the majority (7,358; 59.6%) are larger than 2 cm (referred to below as “large items”) (Fig 5A and S2 Table). There are several indications for onsite knapping (see below). However, the relatively low representation of the small fraction (37.4–44.8% in the different units) suggests that some winnowing and sorting of smaller and lighter artifacts occurred [8,45,46] (see Fig 5A and S2 Table). This post-depositional winnowing was likely caused by agents too weak to have affected the large artifacts (cf. [8,46], and see discussion below).

All four lithic assemblages (Units 3a, 3b, 5a and 5b) are made almost entirely on flint, with only 24 artifacts made of limestone (four cores, thirteen flakes, one retouched tool, two undefined artifacts and four modified limestone pieces [MLP]; see below). A single basalt hammerstone was found in Unit 5b.

There are both lateral (between areas) and vertical (between stratigraphic units) differences in lithic densities (Fig 5B and S3 Table). Refitting sequences in Area A and in Area B (see below) appear to be associated with lower densities of lithic artifacts in a clay loam matrix, while gravely contexts did not contain refitting sequences.

The faunal assemblages found in EQ include 2,030 bones (excluding the 2009–2011 bone assemblage). To date, only 88 (4.3%) were identified to the genus/species level. Hundreds of other specimens that can be assigned to size-class form the basis of an ongoing analysis. Bones were uncovered in all excavation areas and units, although in some areas they were less frequent. For example, in Area C, Unit 5a and Unit 5b include 317 bones from 33 excavated squares, whereas a similar number of excavated squares in Area A yielded 1116 bones, retrieved from two stratigraphic units. As shown by [34], bone preservation and bone frequencies were not affected by post-depositional dissolution, i.e., patterns of bone densities are a reflection of depositional processes.

Several hominin bones, representing three individuals, were found in three of the sedimentary units (Unit 1, Unit 3b and Unit 5a), all associated with MP occupations [33].

### Archaeological remains from Unit 3a

**Fig 6:**
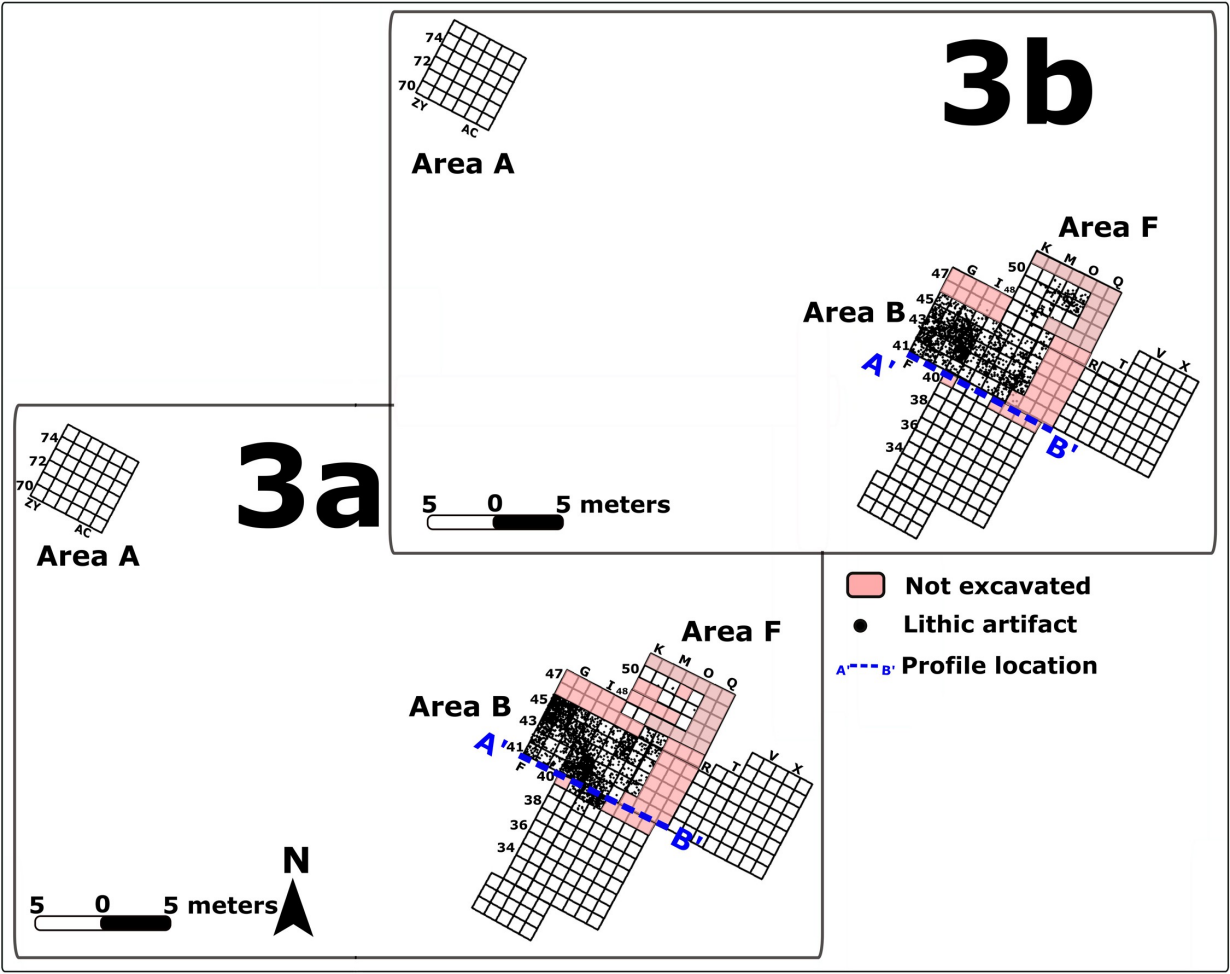
Lateral distribution of lithic artifacts in unit 3a and in unit 3b. Dashed blue line marks the location of the vertical distribtuion of artfiacts shown in S1 Fig. GIS rendition: R. Ekshtain [47].

The archaeological material from this archaeological unit is confined to the top of geological Unit 3a in Area B, at 22.0–21.7 m above mean sea level (amsl) (Fig 6) and in Area F at 21.26–21.2 m amsl. The occupation in these two areas was exposed over a total area of 10m^2^ (Fig 6) and maximum thickness of 30 cm (S1 Fig). The finds were deposited in a silty clay matrix incorporating gravel clusters.

**Fig 7:**
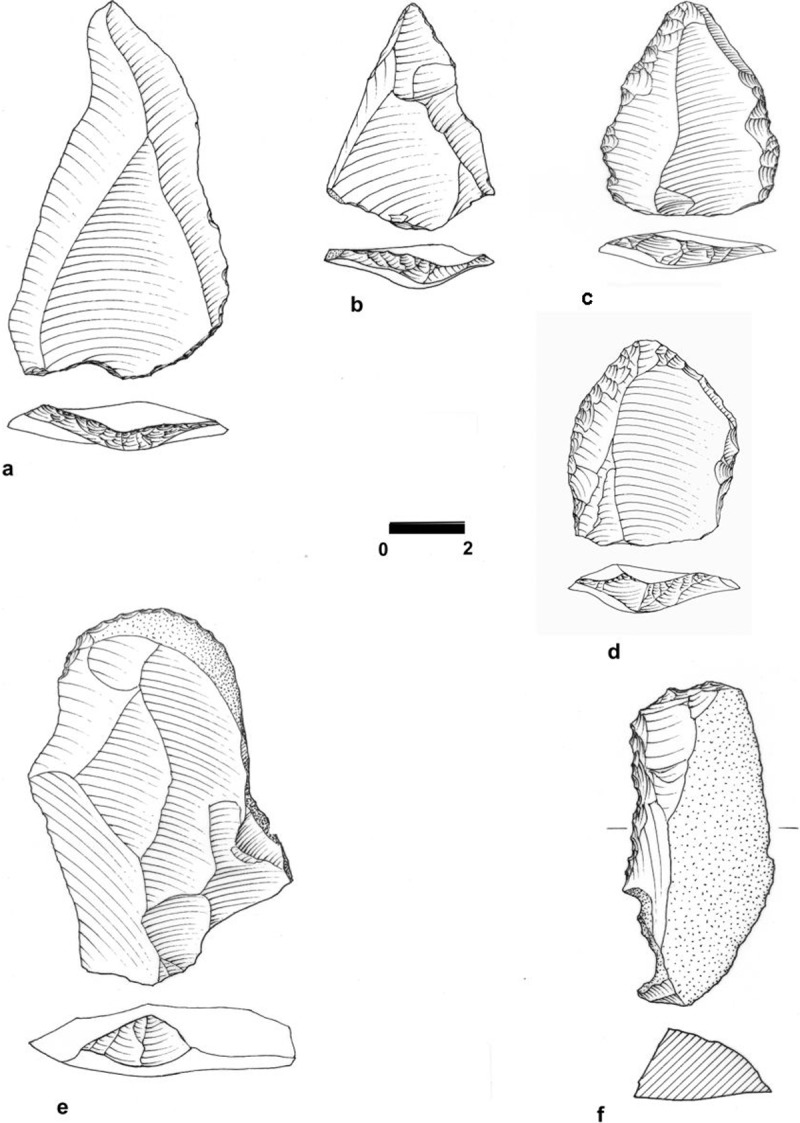
Debitage and tools from EQ. a: Levallois point (#F1844), Unit 3a Area B; b: Levallois point (#F2295), Unit 3a Area B; c: Side-scraper (#F1031), Unit 5b Area A; d: Side-scraper (#F1219), Unit 5a, Area A; e: Atypical end-scraper (#F1594), Unit 3a, Area B; f: A composite tool side- and end0scraper on cortical blade (#F1134), Unit 5b, Area A. With permission from M. Smelansky.

The lithic assemblage from this unit contains 2,262 artifacts (Table a in S2 Table) and lithic artifact density is 211.40/m^3^ (S3 Table). It is dominated by flakes; cores, cortical elements and Core Trimming Elements (CTEs) appear in low frequencies (Table b in S2 Table). This assemblage configuration suggests that part of the material was knapped off-site. Only a small percentage of the items (~10%) is associated with Levallois technology (Fig 7A and 7B). Among the other technological systems encountered in the assemblages are cores-on- flakes (1.5% of the assemblage; n = 33) and blade/bladelet production, suggested by a small number of items (constituting 3%). Among the Levallois elements the frequency of points is low. A small proportion (3.3%) of the artifacts in this unit (debris excluded) was modified by retouch into retouched flakes and blades, side and end-scrapers, notches and denticulates (Fig 7E).

**Fig 8:**
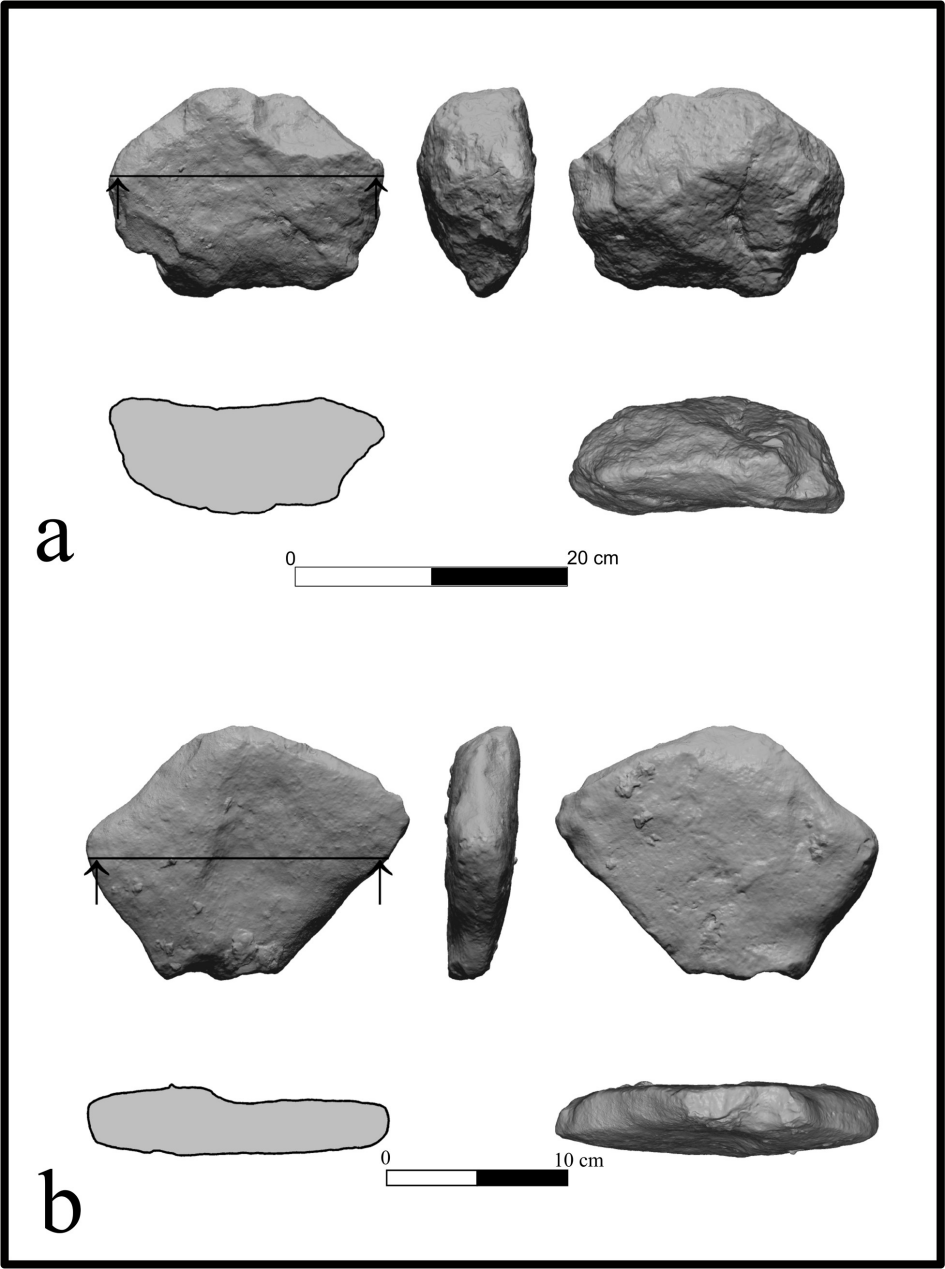
Modified limestone pieces (MLP) from archaeological unit 3a. a. MLP#3, Area B; b. MLP#4, Area F. With permission from the Computerized Archaeology Laboratory, The Institute of Archaeology, The Hebrew University of Jerusalem.

Two large flat limestone slabs bearing signs of modification (Modified Limestone Pieces; MLP) were found in squares K41 (Area B) and N/M49 (Area F). The dimensions of MLP#3 are 198.34X 148.92X 79.65 mm, its circumference is 558 mm and it weighs 2.48 kg. The item was shaped by flaking its edges to create a hierarchy between a relatively flat working surface and the opposite face, which acted as supporting “stand” (Fig 8A). The second MLP (MLP#4) measures 184.91X143.4X40.08 mm, 540 mm in circumference and weighs 1.28 kg. This item is a small slab with a relatively flat face (Fig 8B).

Fifteen bones from Unit 3 were identified to genus/species. Those include seven bones of aurochs (*Bos primigenius*), two of Mesopotamian fallow deer (*Dama mesopotamica*) and two more that are probably of fallow deer, one bone of a wild boar (Sus scofa) and three of mountain gazelle (*Gazella gazella*).

### Archaeological remains from Unit 3b

Human occupation associated with Unit 3b was exposed over a total area of 29.12 m^2^ in Area B at 22.50–22.00 m amsl (50 cm thick) and in Area F at 21.85–21.27 m amsl (~55 cm thick) (Fig 6). The lithic assemblage contains 1,693 artifacts, with an average artifact density of 58.14/m^3^ (S3 Table). The composition of this assemblage is similar to that recovered from Unit 3a, and it is dominated by flakes with low frequencies of cores, CTEs and cortical elements (Table b in S2 Table), suggesting some off-site knapping. Similar to the Unit 3a assemblage, the frequency of Levallois-associated artifacts is low (7%), and points are few (n = 14; 0.8% of the total assemblage). Other technological systems in this unit include cores-on-flakes (1.8% of the assemblage; n = 30) and items that suggest some blade/bladelet production (3.1%). Blanks modified by retouch constitute 4% of the assemblage. The typological composition is similar to Unit 3a archaeological assemblage.

**Fig 9:**
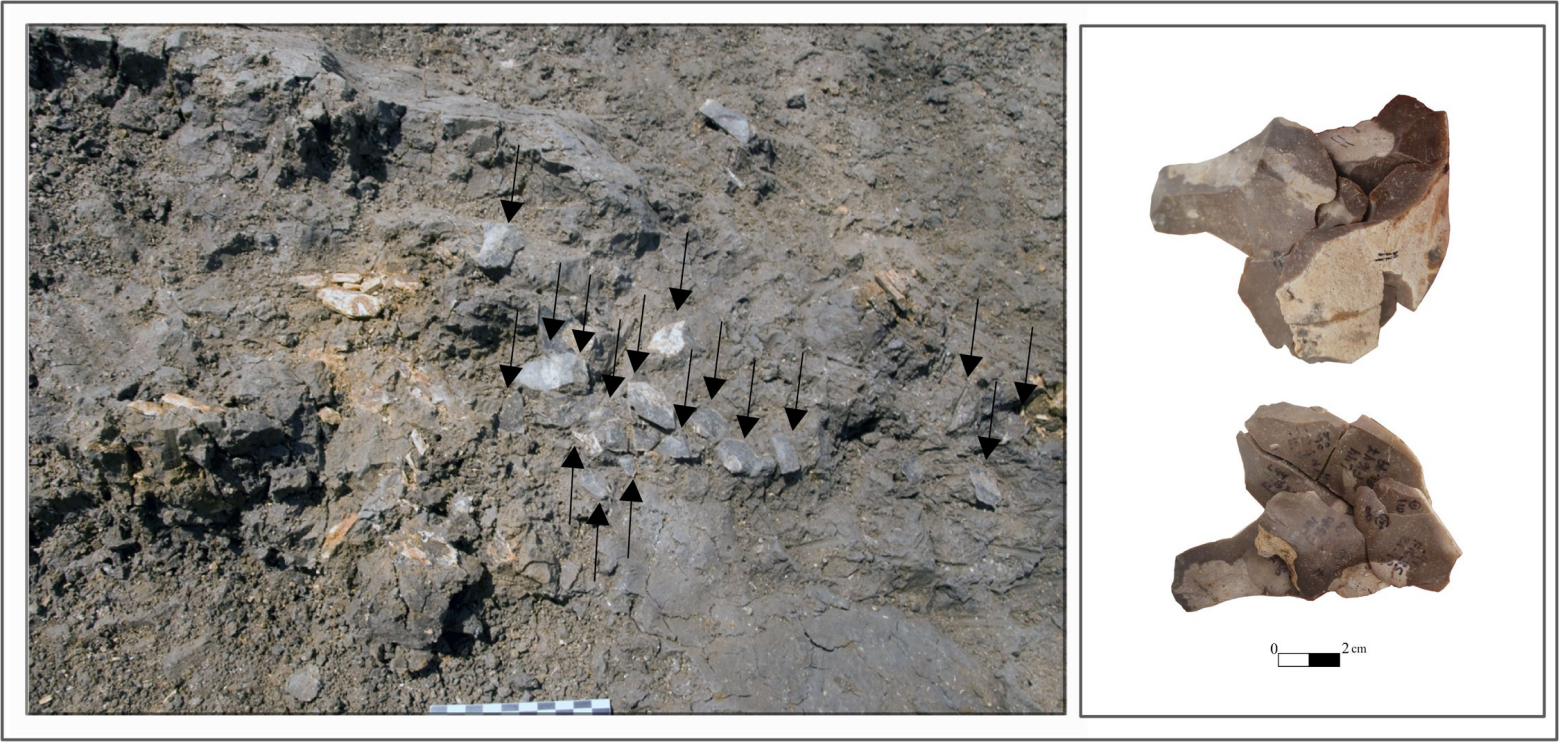
Lithic refits. Left: One of the refitting sequences as seen in the field; Area B Unit 3b. Photo E. Hovers. Right: Part of the refitted knapping sequence (two views of the same aggregate). Photo N. Mitki.

Preliminary refitting attempts yielded four refitted aggregates, composed of 23 flint pieces in total, from Area B (Fig 9). All the artifacts were uncovered from clay loam matrices devoid of pebbles and cobbles. Most of the artifacts were identified in squares L44-L45s, with minor horizontal (1m) or vertical (12 cm, 22.08–21.96 amsl) displacement. All these aggregates are made on the same flint type and seem to belong to one reduction sequence. Aggregate 1 includes a flake and a core (a total of 2 items). Aggregate 2 includes flakes and cortical elements (n = 15). Aggregate 3 includes flakes and CTE (n = 5). The fourth aggregate consists of 2 flakes.

**Fig 10:**
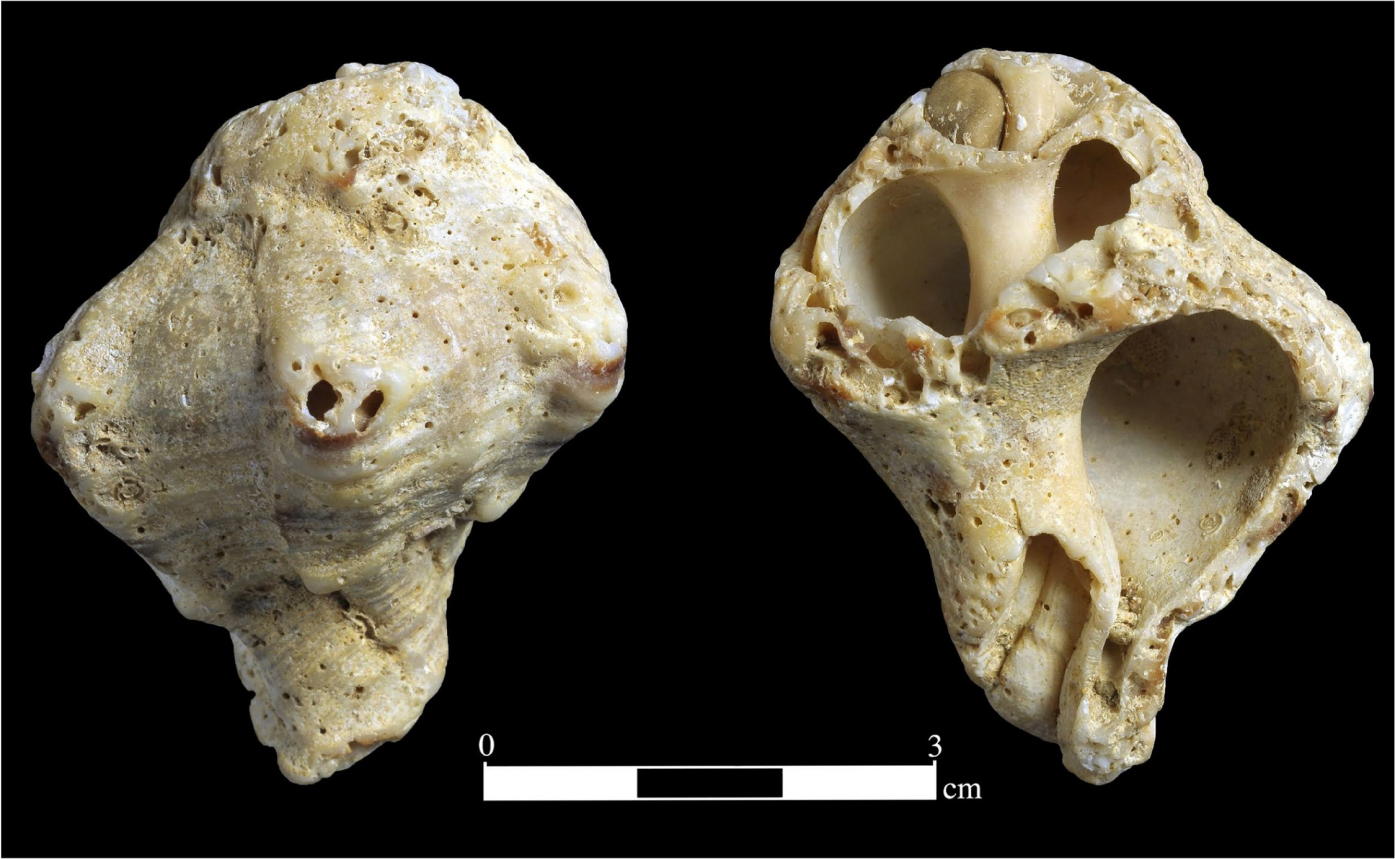
*Hexaplex trunculus* mollusk (#M1095), Unit 3b, Area B. With permission from Clara Amit.

A single gastropod, *Hexaplex trunculus* (Linnaeus, 1758) was found in Area B (square G44, 22.21 m amsl). This is a common Mediterranean gastropod of the family Muricidae and it lives in the intertidal to infra-littoral zone. The taphonomy of the EQ specimen implies it was collected after being washed ashore. Although broken, it currently measures 45.71 mm in height, and 34.24 mm in width. The shell is heavily abraded and broken, its apex and the outer lip are missing, and parts of the other whorls are also broken. It exhibits strong pitting, the result of bio-erosion due to activities of marine invertebrates (worms and clione sponges) on the dead mollusk prior to its washing ashore. In addition, there was a “lace” pattern adhered to the inside of the shell (visible from the aperture), that was identified as *Onychocella* cf. *marioni* [48], a common Mediterranean bryozoan (N. Sokolover, personal communication). A small rolled pebble is stuck in one of the top whorls (Fig 10).

The mollusk could have reached the site only by human transport from the seashore (assumed to have been at the time some 7 to 10 km westward; see above), which makes it a manuport. However, when examined under a binocular microscope (up to x60), no traces of human manipulation similar to those known from other MP sites (e.g., perforation for suspension or pigment traces, cf. [49]), were identified. Whether the shell had a social non-subsistence role or practical function [50] is unknown.

**Fig 11:**
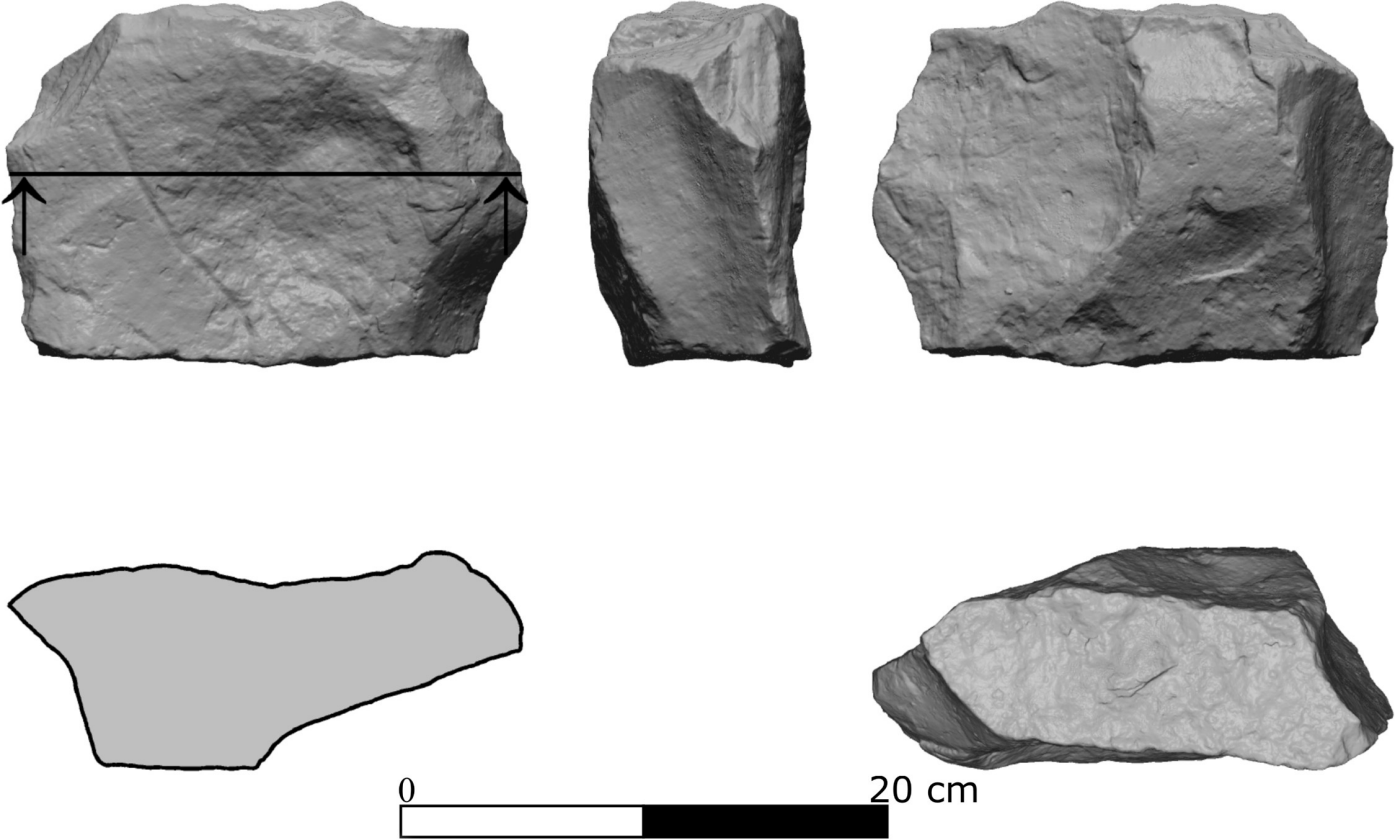
Modified limestone piece (MLP #1), Unit 3b, Area B. With permission from the Computerized Archaeology Laboratory, The Institute of Archaeology, The Hebrew University of Jerusalem.

Two MLP were discovered in archaeological Unit 3b in Area B. One of the items (IMLP#1) was found in square G43 in a dense lithic concentration. Its measurements are 18.69X151.68X83.44 mm, 615 mm in circumference and more than 3 kg in weight. This piece was flaked all around its edges and shaped using the natural properties of the stone to create a relatively flat working surface, at the center of which is a rounded depression (Fig 11). MLP#2 was found in two fragments in Area B. It is flat and irregular in shape (252.22X177.01X106.83 mm, 658.46 mm in circumference; >3 kg).

Twenty-two animal bones were identified to the genus/species level. Those include nine bones of aurochs, two of equid (*Equus* sp.), five of fallow deer, and six of mountain gazelle.

### Archaeological remains from Unit 5a

**Fig 12:**
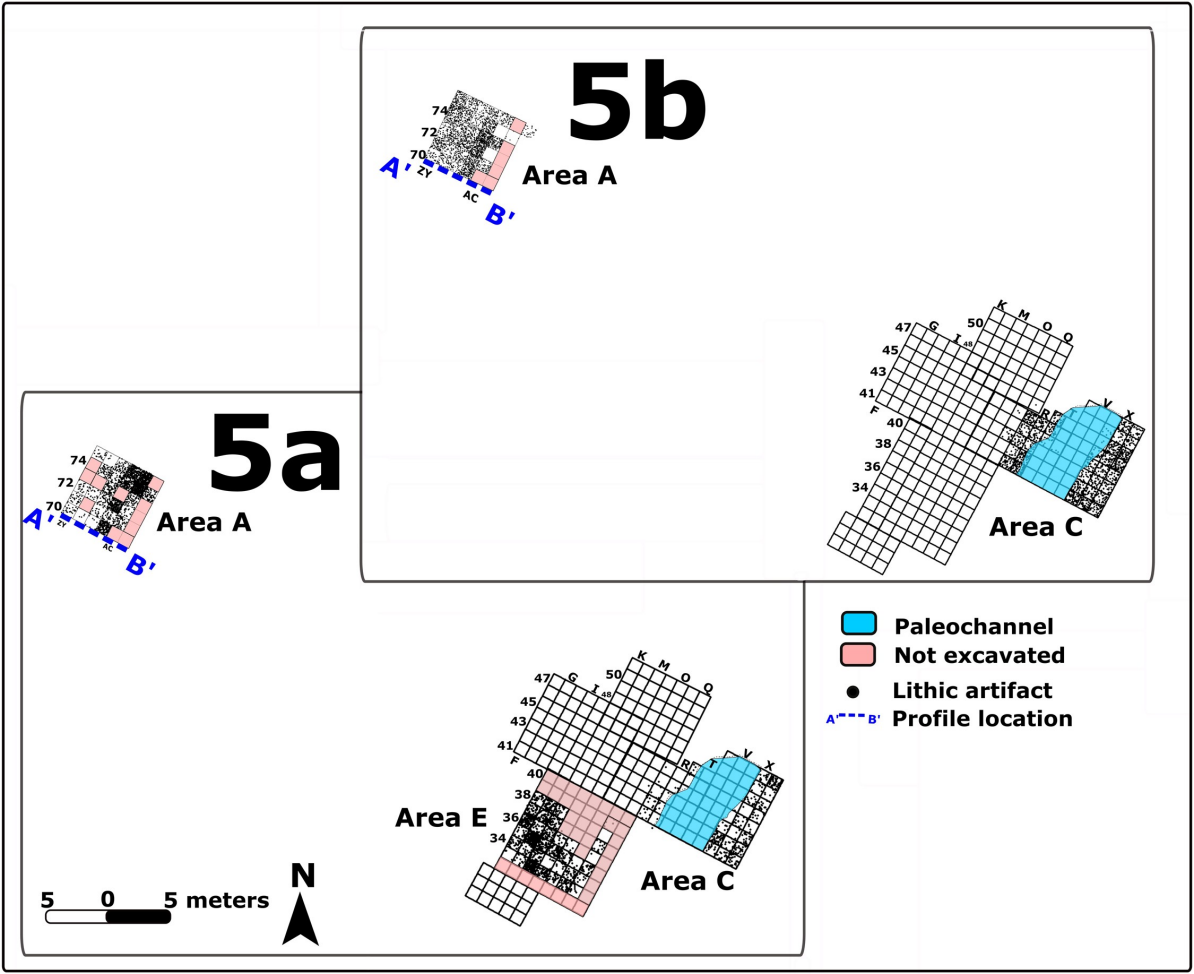
Lateral distribution of lithic artifacts in Units 5a and 5b in Areas A, C and E. Location of a late (Islamic period) channel in Area C shown in blue. The vertical distribution along the marked profile line is shown in S2 Fig GIS rendition: R. Ekshtain [47].

Archaeological remains in Unit 5a are confined to its top part in Area A, at elevations 24.00–23.50 m (50 cm thick) (Figs 12 and S2). They also appear in Area C at elevations 23.70–23.40 and in Area E at elevations 23.70–22. 70 m amsl. Archaeological remains of this unit were encountered also in stratigraphic profiles in Area B but were not excavated. The human occupation associated with Unit 5a was thus exposed over a total area of 70.8 m^2^ (Fig 12), making this the most wide-spread occupation. Unit 5a is also the thickest unit documented at the site (S3 Table), however this is due in part to post-depositional accumulation of pedogenic calcite that increases sediment volume. This process is pronounced in Unit 5 compared to other stratigraphic units [34]. After dissolving calcite in the laboratory and recording sediment volume before and after dissolution, we estimated that the volume of Unit 5a increased by 30% from its initial volume due to the accumulation of calcite nodules. The original volume is therefore estimated to have been 49.5 m^3^. The number of lithic finds in this unit is the highest (n = 4,265). Given the reconstructed increase in sediment volume, the original density of the lithic finds is estimated as 86.2 items/m^3^ (S3 Table).

**Fig 13:**
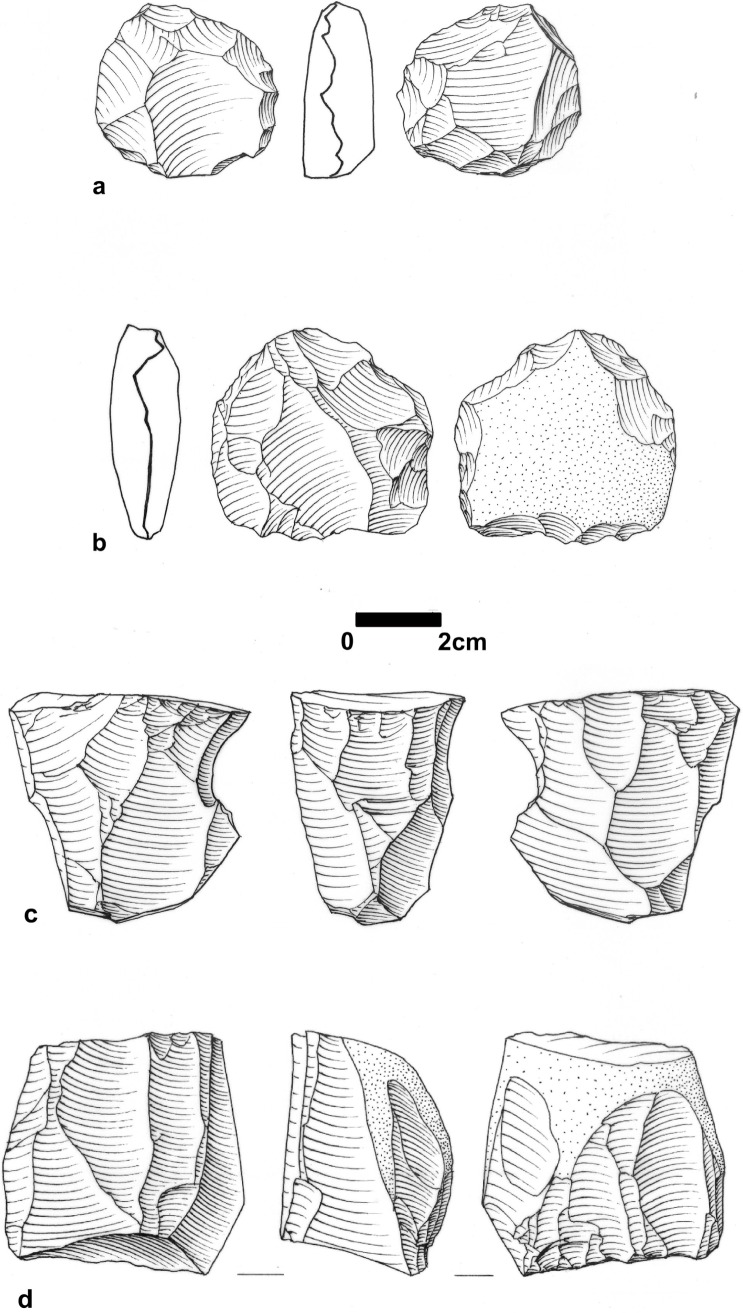
Cores form EQ. a: Levallois core (#F738), Unit 5a Area A; b: Core on flake (#F736), Unit 5a, Area A; c: single platfrom cores for the productions of blades/bladelets (#F743), Unit 5a Area A; d: non Levallois core for blades and flakes (#F1044), Unit 5b, Area B. With permission from M. Smelansky.

The lithic assemblage of Unit 5a is flake dominated. Cores and CTEs occur in low frequencies (Fig 13A–13C) (Table b in S2 Table). Cortical flakes appear in low frequencies similar to the other units. Similarly, frequencies of Levallois items are low (6%) and among them the percentage of points is low. Cores-on-flakes constitute 1.5% (n = 65) of the assemblage, whereas 0.6% are bladelets attesting to an additional technological system for bladelet production (Table b in S2 Table). Retouched blanks constitute 3.1% of the assemblage (Fig 7D), and are mainly retouched flakes and blades, side- and end-scrapers, notches and denticulates.

Eleven refitted aggregates were found in sediments of Unit 5a in Area A, composed of 25 pieces in total. These aggregates represent several short reduction sequences. Five aggregates constitute refits of 2–4 flakes/blades each. An additional five aggregates consist of a core and a detached item (flake, blade or cortical element), and a single aggregate consists of a retouched tool and a flake. In four of the 11 aggregates, refits originated from both Unit 5a and from Unit 5b. The longest vertical displacement recorded in this area is 61 cm while the horizontal movement reached in one case 4 meters, highlighting the scale of movement of artifacts in Area A compared to the earlier units in Area B.

Twenty-four bones were identified to the genus/species level. Those include 17 bones of aurochs (*Bos primigenius*), two of equid (*Equus sp*.), three of fallow deer (*Dama mesopotamica*), and two of mountain gazelle (*Gazella gazella*).

### Archaeological remains from Unit 5b

This is the uppermost MP occupation. It is confined to the lower part of Unit 5b in Area A, at elevations between 24.90–24.20 m amsl (Fig 12) and to Area C at elevations 24.20–23.70 amsl. Archaeological material from this unit was observed also in prfiles of Areas B, E and F, but was not excavated. The total thickness of the unit, complied from the various exposures, was 1.49 m.

The unit was exposed in excavation over a total area of 64 m^2^ in areas A and C (Fig 12). The assemblage includes 4,058 lithics. Based on laboratory assessments (see above), the post-depositional formation of calcite nodules may have increased the original sediment volume by ca. 50%. The original density of the lithic finds is therefore estimated as 221.14/m^3^. This is the densest lithic assemblage at the site (Figs 6 and 12 and S3 Table).

Similar to all other assemblages, it is flake dominated (Table b in S2 Table), while cores and tools are few (Figs 7C and 7F and 13D). Frequencies of CTE and cortical pieces are similar to those in the other units. Levallois elements are few (5%), and the percentage of points is low. The frequency of cores-on flake is low (0.6%; n = 23) and bladelets account for 0.9% of the assemblages. The typological composition of the retouched items is similar to other units.

**Fig 14:**
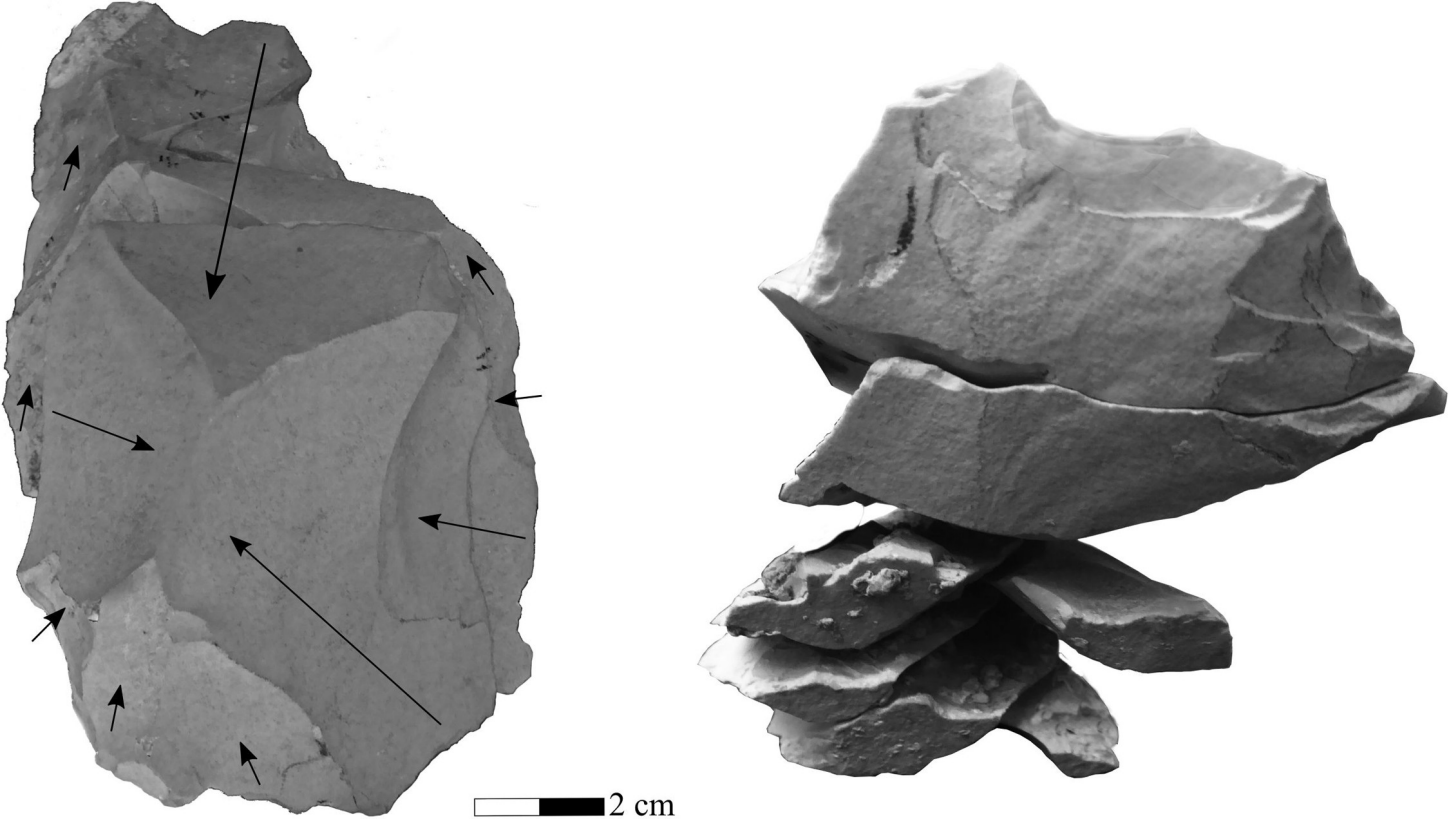
A sequence of refited flakes and blades, Area A. Left—top view, showing the dorsal face of the first flake in this sequence and parts of scars from later flakes in the sequence. Arrows mark the direction of previous removals. Right—side view of the same sequence, showing the sequential removals of flakes from the same striking platfrom of a prepared core. Drawing and photos N. Mitki.

Ten refitted aggregates, totaling 27 items, were found in Area A, representing short reduction sequences. These include five sequences of flakes/blades, three sequences of refitted flakes and cores and two sequences of flakes and cortical pieces or CTE. One aggregate consists of items from both Units 5a and 5b (Fig 14). The longest vertical movement recorded is similar to Unit 5a. The longest refitted sequence (n = 7), made on Cenomanian flint originating from Mt. Carmel, consists of non- cortical shaping flakes with plain striking platforms. It begins with two large flakes and as the knapping continued, blanks became shorter. The core itself was not found, suggesting that the last phases of the knapping sequence may have shifted spatially.

Refitting attempts in Area C have resulted so far in two aggregates. One consists of two flakes and the second of three cortical elements. In this area, the aggregates show little to no horizontal or vertical movement.

Fifteen faunal items identifiable to the genus/species level were found in this occupation unit. Those include ten bones of aurochs, one of equid, one of wild boar, and three of mountain gazelle.

## Hominin remains

The hominin remains found in EQ represent belong to individuals in three distinct stratigraphic units [33]. EQH-1, a non-diagnostic human skull fragment, was recovered from waterlogged sediments of Unit 1 in a geological trench outside the excavation area (Fig 4 in supplementary information of [33]). The second fossil, EQH-2, is an upper third molar from Unit 5a in Area A. Diagnostic traits of the specimen place it with high statistical probability within the Neanderthal population [33].

**Fig 15:**
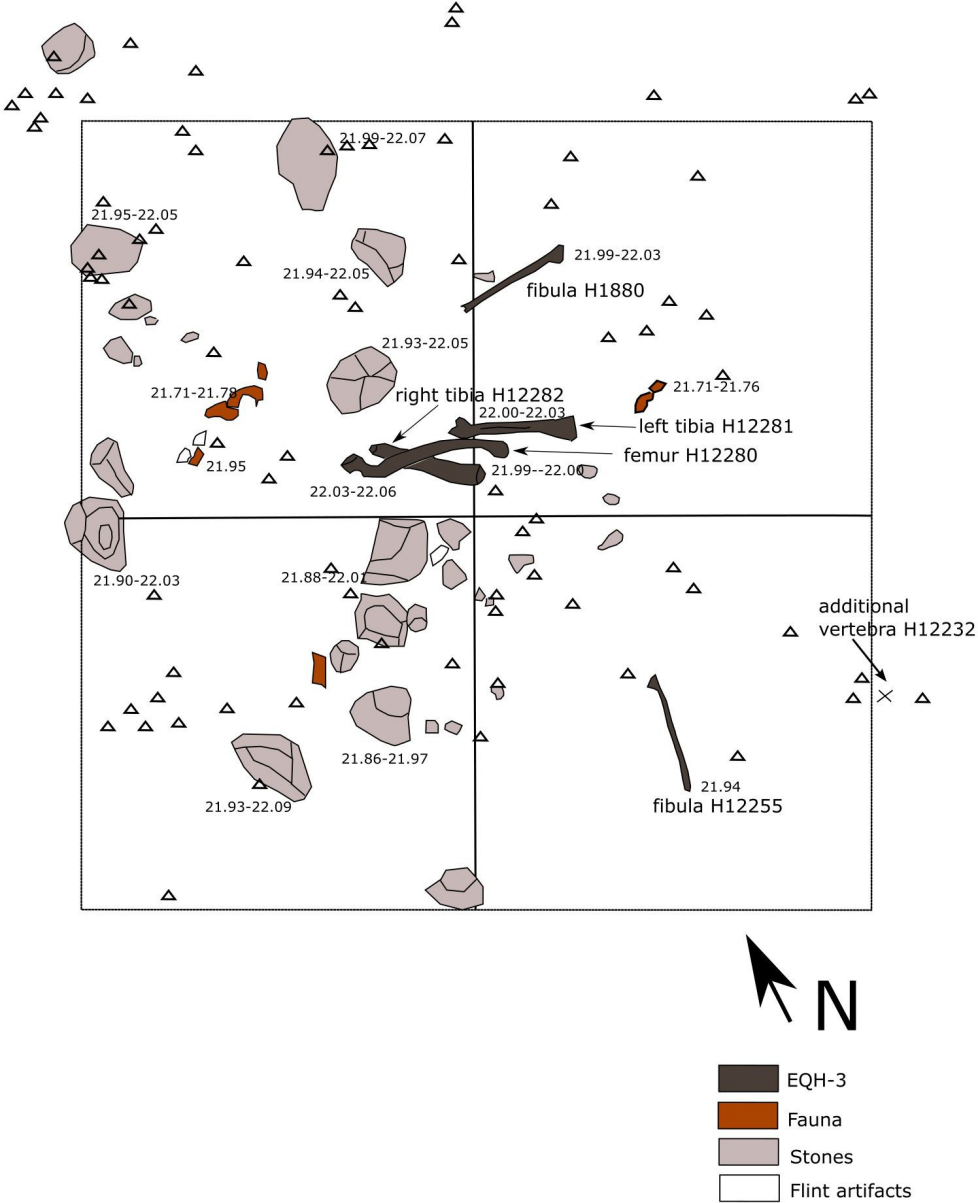
Distribution map of the remains of EQH3. The five lower limb bones of EQH3 (modified form [33] and the location of a sixth bone (marked by X; see description in the text) are shown in relation to non-hominin finds. GIS rendition: R. Ekshtain.

Specimen EQH-3 was found in Area B, Unit 3b. It consists of five lower limb bones—a femur, two tibiae, and two fibulae (Fig 15). A sixth element, a badly preserved part of a human lower lumbar (4^th^ or 5^th^) vertebra, was found at a distance of 0.5 meters to the east of one of the fibulae (Fig 15) and was identified in the laboratory post-excavation. It likely belongs to the individual EQH-3. The bones of EQH-3 were associated with the occupation horizon in Area B (Fig 6), where fresh flint artifacts (including the four refitting aggregates reported above), fragmented animal bones, natural limestone pebbles and cobbles as well as MLP were discovered.

The nearly complete femur and the left tibia of EQH-3 were partially articulated. The bones were aligned along the same axis, with the right tibia parallel to the left. One of the two fibulae (B1880) was discovered ca. 50 cm north of the femur-tibia cluster, and the other fibula (B12255) ca. 70 cm south of the cluster. No duplicate bones were found, suggesting that these bones represent a single individual. Morphometric and computed tomography analyses of the femur and two tibiae (the only bones sufficiently preserved for such analyses) were conducted, the results of which indicate that this is a young Neanderthal male. The individual appears to have suffered from an early age pathology that would have caused limping [33].

## Evidence for the use of fire?

One of the characteristic of Levantine late MP caves, typically identified as residential sites, is the intensive use of fire ([51] and references therein). In MP open-air sites in the Levant, such evidence is less comprehensive or clear. Importantly, not every use of fire or hearth leaves a durable and recognisable footprint in the archaeological record [52]), whereas burnt lithic or faunal remains may be the result of accidental introduction into the fireplace [53,54]. As a result, the relative frequency of heated bones and stones found at most sites, especially in open-air contexts, is expected to be low (e.g. [9,55,56]. In this section we discuss several proxies that may be related to the use of fire in EQ (e.g., geoarchaeological detection of heated sediments, as well as heated stones and bones, and their spatial associations; [55] and references therein, [57–59]).

No *in situ* combustion features were identified during the large-scale excavation at EQ. The faunal remains collected in the 2013 excavation did not show visual evidence of burning (i.e., in coloration, brittleness or fragility), an observation supported by a geochemical study of a comprehensive bone sample from all the excavation areas and all the stratigraphic units, which did not identify any evidence of bone heating or burning [34,38] S3 section). In addition, no signs of fire were found in micromorphological thin sections [34]. However, burnt flint artifacts were identified in all excavation areas, based on the presence of creasing and potlids, two visual properties that are reliable markers of burning (e.g., [55,60]). Excluding other, less secure indications for burnt flint (e.g., color alterations; [61]), these two characteristics help to identify a minimal number of burnt flint items.

**Fig 16:**
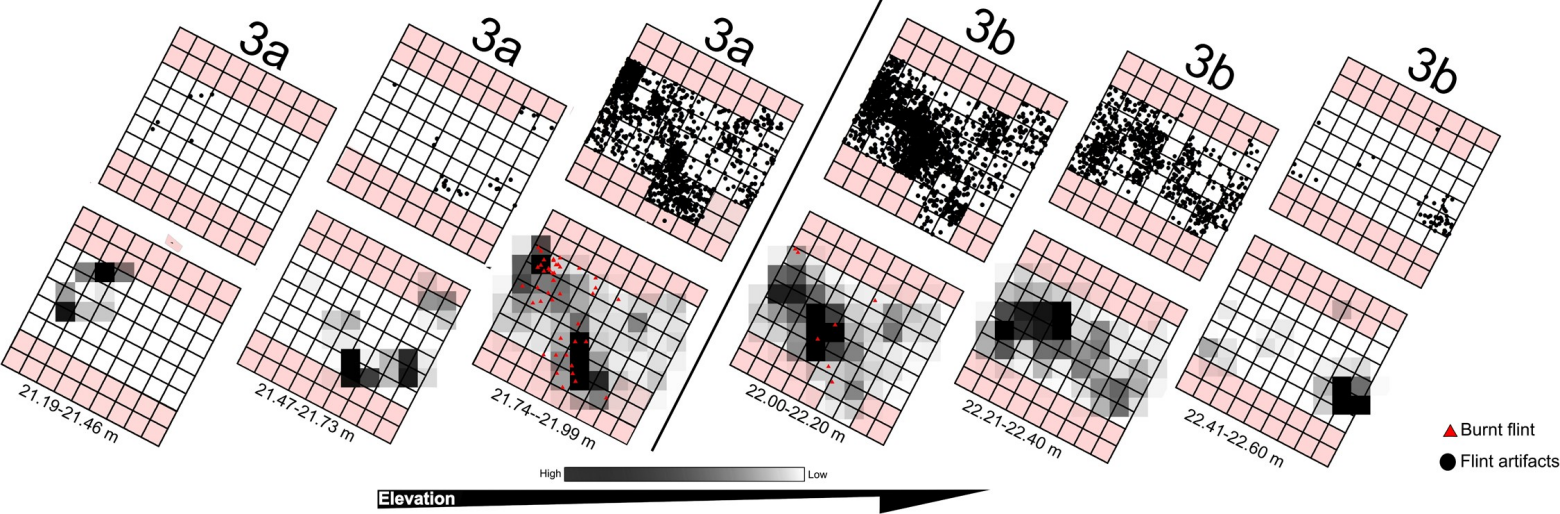
Distributions (top row) and densities (bottom row) of non-burnt and burnt flint artifacts in Area B, Unit 3a and Unit 3b. Kernel density was calculated according to standard deviation using ArcMap 10.3 search radius = 1. Unexcavated areas shown in pink. GIS renditions: R. Ekshtain.

In the small 2009–2011 excavation area, burnt flint items constituted 8.4% across all size classes [8]. In all the archaeology-bearing units of the 2013 excavation a total of 170 burnt flint artifacts >2 cm (1.9% out of the large lithic elements), and 254 <2 cm, (5.1% of the microartifacts) were recovered. Burnt artifacts co-occur both vertically and laterally with the highest densities of lithic artifacts (Fig 16). Given the site’s location in an area subject to ongoing fluvial and alluvial processes, such evidence is not necessarily an indication of *in situ* ‘phantom hearths’ (e.g., [62]), or of the original location of activities. Still, the evidence suggests that fire was used at the site in the context of human occupations. That some spatial re-arrangement occurred due to post-depositional movement would suggest that such putative hearths were altered beyond recognition. Alternately, it is possible that combustion features from whence the burnt artifacts derived were located outside the excavated areas [8,34]. Indeed, Boness and Goren [35] who identified burnt flints and bones in their micromorphological study of samples from the 2009–2011 excavations.

## Discussion

The data presented in this paper provide information about three aspects of hominin behavior at the site of ‘Ein Qashish. The first relates to the activities carried out at the site. The second pertains to the intensity of occupation at the site over time. A third topic of interest is the role of the site in the Levantine late MP settlement pattern.

The OSL dates place human occupations at EQ in late MIS 4 and in MIS 3. Paleoclimatic reconstructions suggest that while temperatures were lower than at present, the general trend of temperature increase [63]. Associated paleorainfall amounts were lower than at present with a general trend of increase in mean annual rainfall [64,65]. The onset of the marsh phases at EQ was correlated to wetter and warmer MIS 3 and MIS 4 phases [42,63], leading to small-scale floods and base flows along the Qishon stream and to possible blocking of the narrow gap by sand. This in turn caused backward inundation. This inland wetland was drained when stream flows of increased flood magnitudes breached the blocking sand [19] during cooler and drier Heinrich events (H6 and H4) associated with low Lake Lisan levels [44]. Isotope values of Unit 3b soil carbonates indicate that they formed under cooler and wetter conditions in comparison to Units 4 and 5 [34,66].

The site extended across at least 1300 square meters [8]. The various occupations existed during the time period of ~71 ka to 54 ka, as defined by the OSL dates. This range can be narrowed to roughly 70–60 ka [8,19,33]. In this study we sampled four archaeological units, each with a relatively large spatial exposure. The human occupation took place concomitantly with cycles of marshes and draining of ephemeral water bodies, interspersed with periods of landscape stabilization, at an interface of marsh and floodplain dynamics. It was suggested that the MP human occupation could only be seasonal [8] since in winter the area was likely flooded repeatedly with seasonal high water stands. Human occupation would be feasible from early summer to early autumn, namely some 6–8 months of the year (see Fig 2). The availability of water in the Qishon stream would make the area especially attractive during the summer.

With the exception of a handful of pollen grains [8], and traces of vegetation growing on stabilized soils [34], no plant remains were preserved in EQ. Based on present-day analogues [67], the riverbank habitats combined with marshes and localized seasonal water bodies suggest a diverse vegetation that likely persisted, at least partially, into the dry summer months and may have been a main attraction for animals and humans. Located in the narrow water gap between Mt. Carmel and the Tiv’on Hills, the landscape around the site made this area suitable for the hunting of large herbivores, using topography to disadvantage prey such as *Bos* (cf. [68–70].

While still preliminary, the faunal analysis shows that all the units contain exclusively ungulate remains dominated by aurochs, similar to the 2009–2011 assemblage [8]. Small game and carnivore skeletal elements are entirely absent from the faunal sample. The four main mammalian species at the site are aurochs, equids, fallow deer and gazelle. These as well as other animals present at the site have diverse habitat preferences. The aurochs’ preferable habitat is extensive dense vegetation intermingled with swamps and river valleys [71]. Wild boar exists in dense wetland environments. Other species, such as mountain gazelle, Mesopotamian fallow deer, the equids, and roe deer represent habitats ranging from grasslands, open parkland and dense woodland [72,73]. Thus, the animal species known from the site reflect an ecological ecotone that was exploited by the site’s dwellers.

### Perspectives on site functions

The unusually large excavated area exposed a range of activities practiced during the different occupations. Three main proxies (knapped lithics, MLP and fauna) are used here to examine the nature and function of EQ and changes through time.

Preliminary comparisons between excavation areas in the same stratigraphic context, as well as between stratigraphic units, show low intra- and inter-assemblage variability. Yet, there are spatial differences within stratigraphic units in lithic densities, faunal presence, and the relative abundance of each category. For example, Area F presents a high density of faunal remains while in Area B lithics are relatively more abundant.

The lithic assemblages constitute the most prolific source of information for possible activities at the site. Throughout time and space, flint constitutes a nearly exclusive raw material used in lithic production (99.7% out of 12,329 large items; Table b in S2 Table), the remaining 0.3% percent being limestone and basalt. The flint originated from several sources at variable distances from the site ([31]; and see below). On-site knapping is evident by the refitted core-and–flake sets as well as by the presence of cores and hammerstones in the assemblages, but the initial stages of reduction are poorly represented.

Lithic production within each unit involved three main technological systems: Levallois [74], cores-on-flakes ([75] and references therein) and bladelet production ([20] and reference therein) (Table b in S2 Table). In addition, expedient lithic production was documented. The lithic assemblages at EQ represent a range of technological options. With the exclusion of the bladelet production, the flaking systems used do not aim to obtain products of particular shape or size. Blank production, core maintenance (attested by refits) and tool discard suggest provisioning of place [76], consistent with a general activity site, despite the relatively low densities of artifacts.

Some 24 artifacts (including MLP in the assemblages) were made of limestone. This raw material was probably collected from nearby channels, where cobbles of suitable sizes for making MLPs were present. The four MLPs were found in Areas B and F in units 3a and 3b. All the items exhibit shared characteristics, such as reworking of the edges and (possibly) working surfaces with depressions or grooves. In other cases, use wear and residue analyses, as well as experimental work on similar limestone or basalt items (e.g., [77,78]) linked pitted anvils to plant processing (e.g. [79,80]), bone processing (e.g. [81,82]), or bipolar lithic production (e.g.[83]). In the absence of evidence for the latter, it is suggested that the MLPs in the various units of EQ were used for processing vegetal material and/or faunal resources.

Linking the MLPs to edible faunal species may be plausible given that percussion marks were documented on the faunal elements of the 2009–2011 bone assemblage ([8]). In contrast, plant remains did not preserve at the site (see above). Notably, evidence from other Levantine sites suggests that Neanderthals relied on edible plants ([84,85]; see [86] and references therein; [87]), even though processing tools were not identified. The functional and behavioral significance of MLPs at EQ remains to be studied through investigating the spatial association of modified bones with the MLPs (e.g., [81]) and through residue and use-wear analyses.

Faunal body part representation is another proxy that can be used to understand site function. Cave sites usually contain bones from whole carcasses of smaller animals and meaty parts (usually of high nutritional value) of larger animals that were selectively transported to the sites ([88], and references therein). At the place of hunting/butchery on the open landscape, one may expect a more complete representation of medium- and large-bodied species [89] or even a “reversed” body part profile that is biased against the exported meaty parts. Preliminary observations suggest that at EQ limb bones were fragmented anthropogenically. These occurrences indicate that humans were responsible for the accumulation of the animal remains, most probably following hunting. The presence of an almost complete skull of *Bos* and associated neck vertebrae in the area excavated in 2009–2011 may support an interpretation of hunting grounds for herbivores [8].

The proxies of hominid fire use at EQ are conflicting. It is possible to identify in open-air contexts signs of fire use other than clear combustion features (e.g., [56]). At EQ no evidence was detected for hearths or combustion features (see [90] for the distinction between the two forms of fire use). The human occupations at EQ took place in muddy surroundings with high moisture, where ash would dissolve quickly [26]. This may in part explain the absence of macroscopic evidence for fireplaces. Still, no burnt bones were identified, either physically or geochemically. The latter phenomenon cannot be attributed to preservation bias [34,38] and demands explanation either through behavior or through formation processes. On the other hand, the presence of burnt artifacts, typically in close association with lithic assemblages in the various stratigraphic units (Fig 16) does suggest fire use at the site by humans.

One complicating factor related to the use of fire in open-air sites are wildfires. In the Mediterranean climatic zone, lightening is the major cause of wildfires [91], typically occurring during winter ([62]:76), and are usually of relatively low temperature and/or last for short periods. Rarer occurrences of high intensity and/or prolonged wildfires exceeding 500°C may cause visible signs of burning (pot-lids, cracking and crazing; [92,93]) in open-air sites where flint was used as raw material. These could mistakenly be ascribed to human agency. At the specific case of EQ, since winter occupation was highly unlikely (see above), the feasibly of wildfire as the cause of lithic burning is low.

Given the variable lines of evidence, we suggest that the locality of EQ was used for different purposes, including raw material provisioning, knapping, possibly procurement and clearly consumption of faunal resources, and, potentially, processing of animal and or/other resources as hinted by the limestone implements. While the remains of EQH-3 could not be attributed with certainty to mortuary practices, it is possible that activities unrelated to subsistence may have taken place during some occupation episodes (e.g., the occupation in Unit 3b), as attested by the presence of the marine mollusk, and knapping, as identified by refitting. Because the clear lateral and vertical associations and the refitted items suggest that the variable finds represent diverse activities on the paleo-landscape, the archaeological units at EQ are interpreted as generalized occupations rather than as palimpsests of successive task-specific occupations.

### Occupation duration and intensity

Estimating the duration and intensity of occupation(s) at EQ is a major challenge. Based on artifact densities, the composition of the lithic assemblages and the refitted sets, we posit that occupations were both ephemeral and repetitive.

Artifact densities in EQ are overall low (Fig 5B and S3 Table). Even the higher densities suggested for the occupation of archaeological Unit 5b are rather sparse (ca. 221 items per cubic meter) in comparison to MP cave sites ([2]: table 7.2;[94]). For example, in Amud cave where the original sediment volume did not go significant compaction due to diagenesis [57], the densities of lithic items was calculated as >1000/ m³ items per 1,000–1,500 TL years. Other open-air sites (Hummal upper layers, [23]; Umm el Tlel, [95]; Quneitra, [18]) exhibit higher densities than EQ. Higher densities of lithic artifacts were observed in several sites due to mostly differential depositional histories (Negev sites: [13,16,96]; Givat Rabi East: [25]; and some of the Nesher Ramla site units: [28]).

The thickness of the archaeological units suggests repeated occupations on a given paleo-landscape (Figs 6 and 12 and S1 and S2). As shown in Fig 16, the low densities of lithic artifacts may indicate short, ephemeral occupations. Unit 4 presents an exceptionally low density of artifacts, which likely were transported sporadically by geogenic or anthropogenic agents into a water body.

Previous studies indicated that raw material was obtained within the daily exploitation territory of the site [31] and knapped locally, with some of the tools produced off-site and brought to EQ as personal gear and maintained at the site [20,31]. The short refitting sets from the 2013 excavation areas consist of only 2–3 pieces and up to only 8 items. Only a few of the refitted items represent early stages of core reduction. This as well as the low percentage of cortical elements (Table a in S2 Table) in the lithic assemblages suggests that initial knapping typically occurred off-site, while core maintenance took place at the site. These lines of evidence suggest a mobile toolkit.

The ephemeral character in combination with a high rate of sedimentation and local depositional conditions may explain the invisibility of fire residues (see [52] for a formal model of this scenario). In Levantine caves, high frequencies of burnt bones stem from repeated use of hearths in the same places within the cave [94,97], causing charring of pre-existing bone refuse, high bone fragmentation sometimes accompanied by cementation of large amounts of ash (e.g., [57,88,98,99]. In MP open-air sites, there are no natural or built boundaries of the human settlement, therefore the location of fireplaces may have shifted spatially from one episode of occupation to another. If such was the case at EQ, the signatures of fire use would be lost to the archaeological eye.

To summarize, the sedimentary and archaeological records at EQ represent minimally four short and repetitive human occupations during a time span of several thousand years. Importantly, the areas exposed in the excavations were not the only ones occupied, as we estimated the site area at minimally 1300 square meters [8]. This persistent use of the site suggests a special attraction to the particular point, perhaps due to its location on the landscape, in the narrow corridor between the Carmel flank and the Tiv’on Hills near a source of fresh water, for the purpose of monitoring and hunting animals. We suggest that the repeated use of this location present the retention of memory from one occupation phase to the other. EQ seems to have been a long-standing focal point within the settlement system of the late Levantine MP in northern Israel.

### EQ within its broad settlement system

EQ is geographically close to major late MP cave sites in northern Israel. The closest MP occupation is ca. 3 km away in Raqefet cave [100]. Tabun Cave is located 10 km to the southwest, and the Atlit Railway Bridge site [101] is located some 17 km away in the same direction. Kebara Cave is situated ca. 25 km southwest and Amud and Shovakh Caves are located some 45 km northeast in Nahal Amud. The open-air sites Nahal Mahanayeem Outlet and Quneitra are located ca. 60 km and 85 km to the northeast, respectively (Fig 1). The case study of EQ allows us to re-examine the structure of late MP settlement patterns.

Two main areas of lithic raw material procurement were identified [31]. The main one, from which the bulk of the lithic material originates, is located west of the site (on Mt. Carmel). The second procurement area is found southwest of the site in the Menashe Hills, where several flint types were obtained, mostly from secondary sources. High quality, abundant flint sources are located east of EQ but appear to not be imported into the area of the site sampled during the 2009–2011 project [31]. A minute portion of artifacts in the much larger 2013 assemblage was attributed to sources in the latter area, perhaps due to the larger sample size. In general, EQ inhabitants focused on local raw material sources found within daily exploitation territories around the site. Few artifacts were brought as personal gear from more distant sources.

Another item that was brought to the site from beyond the daily exploitation territory is the single *Hexaplex* mollusk. Some of the large ungulate species may have been brought from areas beyond the immediate flood plain and marshes of EQ. Dense vegetation around the site likely prohibited the presence of mountain gazelle and equid, which were most likely procured beyond the immediate surrounding of the locality.

The exploited territory around EQ may have been shared with the occupants of other (broadly contemporaneous) nearby late MP cave sites. For example, the areas of Menashe Hills and Mt. Carmel were probably used as flint procurement areas by the inhabitants of Kebara Cave [102]. Some characteristics of the lithic assemblages are similar between Kebara and EQ (e.g., core-on flakes) [103,104]. Others (e.g., the occurrence of a bladelet reduction system at EQ and the paucity of points compared to Kebara) differ. EQ shared similarities in the lithic assemblage also with penecontemporaneous sites further away (e.g., Amud, Quneitra, NMO, Umm el Tlel and Hummal), suggesting that groups with similar economic goals, knowledge, and mobility strategies repeatedly inhabited the area Thus these sites can be studied as components of integrated settlement systems [18,105–107].

It has been suggested that the occupants of late MP sites in the Mediterranean zone of the Levant practiced residential mobility in relatively small areas around cave sites, resulting in many returns to any given location [2,94,107–111]. Caves were selected for use based on shelter properties and ease of access [112] as well as proximity to ecotones, and were used for varied and generalized functions characterizing social groups, including flint knapping, food processing, mortuary practices and information transfers (e.g., Amud, Kebara caves) [51,113–115]. Many of the open-air sites were interpreted as locations where a narrower range of activities was carried out, often related to hunting (e.g., NMO;[27] Umm El Tlel;[21,95,116] and butchering (Quneitra; [81]; see [96] about sites in the Negev; but see [117]), and as short-term occupations. The absence of physical boundaries in such sites reduces the odds of discovering superimposed anthropogenic deposits similar to those found in caves. Where such repeated occupation events could be identified, open-air sites sometimes demonstrate changes in site functions (e.g., [102,118]).

An increasing interest in open-air sites and an overall change in the perception of the MP has led researchers to the understanding that both caves and open-air sites should be studied as components of integrated settlement systems. In the late MP in particular, it has become clear that open-air sites and caves found in the same geographical vicinity are penecontemporaneous and may represent complementary location choices within a settlement system. This allows us to identify the hierarchy among site locations in the settlement systems of this period. While caves retain their place as first and foremost ‘home base’ locations, the diverse characteristics of open-air sites represent a continuum of roles from task-specific to more generalized ‘home bases’ [10]. This in turn implies that residential mobility during the late MP included also logistical visits between sites (open-air sites and caves). While linking specific caves and open-air sites based on archaeological finds alone is currently unattainable (also due to precision constraints of dating methods), it may not be a necessary step for reconstructing the bigger picture of decision-making criteria and behaviors.

The case of EQ provides an intermediate example between the two polarizing scenarios of site typologies. The site represents a series of consecutive ephemeral occupations, within each of which a range of activities were carried out. Throughout the time of its occupation, EQ functioned mainly as a residential site in which general activities took place, indicating a stable settlement system during the late MP.

## Concluding remarks

EQ includes minimally four diachronic occupations and potentially more than one occupation on a given landscape. At least some of the occupations were short-lived and underwent rapid burial. Unlike other stratified open-air sites currently known from the southern Levant, the series of occupations at EQ does not show drastic changes in site function or in activities carried out. Rather, this locale seems to have been used repeatedly as a generalized residential site (‘home base’), albeit occupations were of an ephemeral nature.

Open-air sites are an integral part of the settlement systems in the Levant during the MP. Together with cave sites, they constitute complementary components of settlement/mobility systems. The case of EQ demonstrates that the rigid dichotomy between ‘home base’ sites of long duration, typically associated with sheltered locales, and task-specific, short-term occupations on the open landscape, is an oversimplification of a complex behavioral system. The challenge for the next phase of research is to come up with analytical and methodological tools to address such complex past realities and to delineate the range of behaviors and processes that may have created them.

## Supporting information

S1 section Site stratigraphy and sedimentology. (DOCX)

S2 section Calcic soils. (DOCX)

S3 section Bones analysis. (DOCX)

S1 Table Laboratory data and OSL ages for ‘Ein Qashish. S2A Table.
(DOCX)

S2 Table (a) The Lihtic assemblages of ‘Ein Qashsi according to stratigraphic units and excavation areas. (b) Technological breakdown of EQ assemblages by archaoelogical units.
(DOCX)

S3 Table Artifact densities in the archaeological units. (DOCX)

S1 Fig Vertical distribution of lithic artifacts in area B unit 3a and in unit 3b. For profile location see Fig 6. GIS rendition: R. Ekshtain.
(TIF)

S2 Fig Vertical distribution of lithic artifacts in area A unit 5a and in 5b. For profile location see Fig 12. GIS rendition: R. Ekshtain.
(TIF)

## References

[1] Sharon G, Zaidner Y, Hovers E. Opportunities, problems and future directions in the study of open-air Middle Paleolithic sites. Quat Int. 2014;331: 1–5. 10.1016/j.quaint.2014.03.055

[2] Hovers E. The Lithic Assemblages of Qafzeh Cave. New York: Oxford University Press; 2009.

[3] Sackett JR. Straight archaeology French-style: the phylogenetic paradigm in historic perspective In: Clark GA, editor. Perspectives on the past: theoretical biases in Mediterranean hunter-gatherer research. Philadelphia: University of Pennsylvania; 1991 pp. 109–139.

[4] Bar Yosef O, Goren N. Afterthoughts Following Prehistoric Surveys in the Levant. Isr Explor J. 1980;30: 1–16.

[5] Bailey G. Time perspectives, palimpsests and the archaeology of time. J Anthropol Archaeol. 2007;26: 198–223. 10.1016/j.jaa.2006.08.002

[6] Dibble HL, Chase PG, McPherron SP, Tuffreau A. Testing the Reality of a “Living Floor” with Archaeological Data. Am Antiq. 1997;62: 629–651. 10.2307/281882

[7] Enloe JG. Geological processes and site structure: Assessing integrity at a Late Paleolithic open-air site in northern France. Geoarchaeology. 2006;21: 523–540. 10.1002/gea.20122

[8] Hovers E, Ekshtain R, Greenbaum N, Malinsky-Buller A, Nir N, Yeshurun R. Islands in a stream? Reconstructing site formation processes in the late Middle Paleolithic site of ‘Ein Qashish, northern Israel. Quat Int. 2014;331: 216–233. 10.1016/j.quaint.2014.01.028

[9] Pop E, Kuijper W, Hees E van, Smith G, García-Moreno A, Kindler L, et al Fires at Neumark-Nord 2, Germany: An analysis of fire proxies from a Last Interglacial Middle Palaeolithic basin site. J Field Archaeol. 2016;41: 603–617. 10.1080/00934690.2016.1208518

[10] Hovers E. Middle Palaeolithic Open-Air Sites In: Enzel Y, Bar-Yosef O, editors. Quaternary of the Levant Environments, Climate Change, and Humans. Cambridge: Cambridge University Press; 2017 pp. 593–601.

[11] Fleisch SJ. Les habitats du Paléolithique moyen a Naam6 (Liban). Bull Musee Beyrouth. 1970;23: 25–98.

[12] Ronen A. Tirat Carmel: A Mousterian Open-Air Site in Israel. Tel Aviv: Institute of Archaeology, Tel Aviv University; 1974.

[13] Crew. The Mousterian Site of Rosh Ein Mor In: Marks AE, editor. Prehistory and Paleoenvironments in the Central Negev, Israel. 1976 pp. 75–112.

[14] Marks AE, editor. Prehistory and Palaeoenvironments in the Central Negev, Israel, vol. 1 Dallas: SMU Press; 1976.

[15] Marks AE, editor. Prehistory and Palaeoenvironments in the Central Negev, Israel, vol. 2 Dallas: SMU Press; 1977.

[16] Munday FC. Nahal Aqev (D35): A Stratified, Open-Air Mousterian Occupation in the Avdat/Aqev Area In: Marks AE, editor. Prehistory and Palaeoenvironments in the Central Negev, Israel, Vol II: The Avdat/Aqev Area, part 2, and the Har Harif. Dallas: SMU Press; 1977 pp. 35–60.

[17] Gilead I, Grigson C. Fara II: A Middle Paleolithic Open-Air Site in the Northern Negev, Israel. Proc Prehist Soc. 1984;50: 71–97.

[18] Goren-Inbar N, editor. Quneitra: A Mousterian Site on the Golan Heights Jerusalem: Qedem vol. 31. Monographs of the Institute of Archaeology, The Hebrew University of Jerusalem; 1990.

[19] Greenbaum N, Ekshtain R, Malinsky-Buller A, Porat N, Hovers E. The stratigraphy and paleogeography of the Middle Paleolithic open-air site of ‘Ein Qashish, Northern Israel. Quat Int. 2014;331: 203–215. 10.1016/j.quaint.2013.10.037

[20] Malinsky-Buller A, Ekshtain R, Hovers E. Organization of lithic technology at ‘Ein Qashish, a late Middle Paleolithic open-air site in Israel. Quat Int. 2014;331: 234–247. 10.1016/j.quaint.2013.05.004

[21] Boëda E, Griggo C, Noël-Soriano S. Différents modes d’occupation du site d’Umm el Tlel au cours du paléolithique moyen (El Kowm, Syrie centrale). Paléorient. 2001; 13–28.

[22] Tensorer Jean‐Marie Le Jagher Reto, Philippe Rentzel, Thomas Hauck, Kristin Ismail‐Meyer, Christine Pümpin, et al Long‐term site formation processes at the natural springs Nadaouiyeh and Hummal in the El Kowm Oasis, Central Syria. Geoarchaeology. 2007;22: 621–640. 10.1002/gea.20177

[23] Hauck TC. Mousterian technology and settlement dynamics in the site of Hummal (Syria). J Hum Evol. 2011;61: 519–537. 10.1016/j.jhevol.2011.01.014 21890177

[24] Wojtczak D. Hummal (Central Syria) and its eponymous industry In: Le Tensorer J-M, Jagher R, Otte M, editors. The Lower and Middle Paleolithic in the Middle East and neighbouring regions. Liège: ERAUL 126; 2011 pp. 289–308.

[25] Ekshtain R, Barzilai O, Inbar M, Milevski I, Ullman M. Givat Rabi East, a New Middle Paleolithic Knapping Site in the Lower Galilee (Israel). Paléorient. 2012;37: 107–122.

[26] Friesem DE, Zaidner Y, Shahack-Gross R. Formation processes and combustion features at the lower layers of the Middle Palaeolithic open-air site of Nesher Ramla, Israel. Quat Int. 2014;331: 128–138. 10.1016/j.quaint.2013.03.023

[27] Sharon G, Oron M. The lithic tool arsenal of a Mousterian hunter. Quat Int. 2014;331: 167–185. 10.1016/j.quaint.2013.10.024

[28] Zaidner Y, Frumkin A, Porat N, Tsatskin A, Yeshurun R, Weissbrod L. A series of Mousterian occupations in a new type of site: The Nesher Ramla karst depression, Israel. J Hum Evol. 2014;66: 1–17. 10.1016/j.jhevol.2013.06.005 24210611

[29] Crouvi O, Barzilai O, Goldsmith Y, Amit R, Matskevich Z, Porat N, et al Middle to late Pleistocene shift in eolian silts contribution into Mediterranean soils at the fringe of the Negev loess, Israel. Quat Sci Rev. 2018;191: 101–117. 10.1016/j.quascirev.2018.04.030

[30] Hovers E, Buller A, Ekshtain R, Oron M, Yeshurun R. ‘Ein Qashish—A New Middle Paleolithic Open-Air Site in Northern Israel. Mitakufat Haeven. 2008;38: 7–40.

[31] Ekshtain R, Malinsky-Buller A, Ilani S, Segal I, Hovers E. Raw material exploitation around the Middle Paleolithic site of ‘Ein Qashish. Quat Int. 2014;331: 248–266. 10.1016/j.quaint.2013.07.025

[32] Barzilai O, Malinsky-Buller A, Ekshtain R, Hovers E. `En Qhasish. Hadashot Arkheologiot. 2015;127 Available: 127 http://www.hadashot-esi.org.il

[33] Been E, Hovers E, Ekshtain R, Malinski-Buller A, Agha N, Barash A, et al The first Neanderthal remains from an open-air Middle Palaeolithic site in the Levant. Sci Rep. 2017;7: 2958 10.1038/s41598-017-03025-z 28592838 PMC5462778

[34] Stahlschmidt MC, Nir N, Greenbaum N, Zilberman T, Barzilai O, Ekshtain R, et al Geoarchaeological Investigation of Site Formation and Depositional Environments at the Middle Palaeolithic Open-Air Site of ‘Ein Qashish, Israel. J Paleolit Archaeol. 2018;1: 32–53. 10.1007/s41982-018-0005-y

[35] Boness D, Goren Y. Site formation processes at the Late Middle Palaeolithic site of ‘Ein Qashish: a micromorphological study. J Isr Prehistory Soc. 2016;46: 5–19.

[36] Sivan D, Greenbaum N. Middle-late Quaternary wetlands along the coastal plain of Israel In: Enzel Y, Bar-Yosef O, editors. Quaternary of the Levant: Environments, Climate Change, and Humans. Cambridge: Cambridge University Press; 2017 pp. 447–456.

[37] Sivan D, Greenbaum N, Cohen-Seffer R, Sisma-Ventura G, Almogi-Labin A. The origin and disappearance of the late Pleistocene–early Holocene short-lived coastal wetlands along the Carmel coast, Israel. Quat Res. 2011;76: 83–92. 10.1016/j.yqres.2011.04.006

[38] Nir N. Bones and formation processes in the Middle Paleolithic open-air site of Ein Qashish. MA Thesis, The Hebrew University. 2016.

[39] Simona Avnaim‐Katav, Ahuva Almogi‐Labin, Amir Sandler, Dorit Sivan, Naomi Porat, Ari Matmon. The chronostratigraphy of a quaternary sequence at the distal part of the Nile littoral cell, Haifa Bay, Israel. J Quat Sci. 2012;27: 675–686. 10.1002/jqs.2537

[40] Salamon A, Zaslavski Y, Shtibelman V, Rockwell T. Earthquake risk at the Nesher Factory, Haifa Bay Area, Israel. Isr Geol Soc Annu Meet. 2000;

[41] Zviely D, Sivan D, Ecker A, Bakler N, Rohrlich V, Galili E, et al Holocene evolution of the Haifa Bay area, Israel, and its influence on ancient tell settlements. The Holocene. 2006;16: 849–861. 10.1191/0959683606hol977rp

[42] Enzel Y, Amit R, Dayan U, Crouvi O, Kahana R, Ziv B, et al The climatic and physiographic controls of the eastern Mediterranean over the late Pleistocene climates in the southern Levant and its neighboring deserts. Glob Planet Change. 2008;60: 165–192. 10.1016/j.gloplacha.2007.02.003

[43] Torfstein A, Goldstein SL, Stein M, Enzel Y. Impacts of abrupt climate changes in the Levant from Last Glacial Dead Sea levels. Quat Sci Rev. 2013;69: 1–7. 10.1016/j.quascirev.2013.02.015

[44] Yaroshevich A, Bar-Yosef O, Boaretto E, Caracuta V, Greenbaum N, Porat N, et al A Unique Assemblage of Engraved Plaquettes from Ein Qashish South, Jezreel Valley, Israel: Figurative and Non-Figurative Symbols of Late Pleistocene Hunters-Gatherers in the Levant. PLOS ONE. 2016;11: e0160687 10.1371/journal.pone.0160687 27557110 PMC4996494

[45] Schick KD. Stone Age sites in the making: experiments in the formation and transformation of archaeological occurrences. Oxford: B.A.R.; 1986.

[46] Malinsky-Buller A, Grosman L, Marder O. A case of techno-typological lithic variability & continuity in the late Lower Palaeolithic. Farming. 2011;2011: 1–32. 10.3828/bfarm.2011.1.3

[47] Ekshtain R. ‘Ein Qashish [Internet]. OSF; 19 May 2019; Available: doi:10.17605/OSF.IO/AMGQ7

[48] Jullien D. Dragages du Travailleur: Bryozoaires espèces draguées dans l’océan Atlantique. 1882.

[49] Bar-Yosef Mayer DE, Vandermeersch B, Bar-Yosef O. Shells and ochre in Middle Paleolithic Qafzeh Cave, Israel: Indications for modern behavior. J Hum Evol. 2009;56: 307–314. 10.1016/j.jhevol.2008.10.005 19285591

[50] Bar-Yosef Mayer DE. The Exploitation of Shells as Beads in the Palaeolithic and Neolithic of the Levant. Paléorient. 2005;31: 176–185.

[51] Hovers E, Belfer-Cohen A. On Variability and Complexity: Lessons from the Levantine Middle Paleolithic Record. Curr Anthropol. 2013;54: S337–S357. 10.1086/673880

[52] Sorensen AC, Scherjon F. fiReproxies: A computational model providing insight into heat-affected archaeological lithic assemblages. PLOS ONE. 2018;13: e0196777 10.1371/journal.pone.0196777 29768454 PMC5955532

[53] Aldeias V, Dibble HL, Sandgathe D, Goldberg P, McPherron SJP. How heat alters underlying deposits and implications for archaeological fire features: A controlled experiment. J Archaeol Sci. 2016;67: 64–79. 10.1016/j.jas.2016.01.016

[54] Mallol C, Hernández CM, Cabanes D, Sistiaga A, Machado J, Rodríguez Á, et al The black layer of Middle Palaeolithic combustion structures. Interpretation and archaeostratigraphic implications. J Archaeol Sci. 2013;40: 2515–2537. 10.1016/j.jas.2012.09.017

[55] Alperson-Afil N, Richter D, Goren-Inbar N. Evaluating the intensity of fire at the Acheulian site of Gesher Benot Ya’aqov—Spatial and thermoluminescence analyses. PLOS ONE. 2017;12: e0188091 10.1371/journal.pone.0188091 29145432 PMC5690626

[56] Hérisson D, Locht J-L, Auguste P, Tuffreau A. Néandertal et le feu au Paléolithique moyen ancien. Tour d’horizon des traces de son utilisation dans le Nord de la France. L’Anthropologie. 2013;117: 541–578. 10.1016/j.anthro.2013.10.002

[57] Shahack-Gross R, Ayalon A, Goldberg P, Goren Y, Ofek B, Rabinovich R, et al Formation processes of cemented features in karstic cave sites revealed using stable oxygen and carbon isotopic analyses: A case study at at Middle Paleolithic Amud Cave, Israel. Geoarchaeology. 2008;23: 43–62.

[58] Stiner MC, Kuhn SL, Surovell TA, Goldberg P, Meignen L, Weiner S, et al Bone preservation in Hayonim Cave (Israel): a macroscopic and mineralogical study. J Archaeol Sci. 2001;28: 643–59.

[59] Hlubik SK. Finding Prometheus: evidence for fire in the early pleistocene at FxJj20 AB, Koobi Fora, kenya. [Internet]. Rutgers University—School of Graduate Studies. 2018 10.7282/T3571G7C

[60] Richter D, Alperson‐Afil N., Goren‐Inbar N. Employing tl methods for the verification of macroscopically determined heat alteration of flint artefacts from palaeolithic contexts. Archaeometry. 2011;53: 842–857. 10.1111/j.1475-4754.2010.00581.x

[61] Sergant J, Crombé P, Perdaen Y. The ‘invisible’ hearths: a contribution to the discernment of Mesolithic non-structured surface hearths. J Archaeol Sci. 2006;33: 999–1007. 10.1016/j.jas.2005.11.011

[62] Alperson-Afil N, Goren-Inbar N. The Acheulian Site of Gesher Benot Ya’aqov Volume II: Ancient Flames and Controlled Use of Fire [Internet]. Springer Netherlands; 2010 Available: //www.springer.com/gp/book/9789048137640

[63] Keinan J. Paleo-environment of the Northern Jordan Rift region based on speleothems from Zalmon Cave, Israel. MA thesis, Department of Geology Institute of Earth Sciences Faculty of Mathematics and Natural Sciences The Hebrew University of Jerusalem. 2016.

[64] Affek HP, Bar-Matthews M, Ayalon A, Matthews A, Eiler JM. Glacial/interglacial temperature variations in Soreq cave speleothems as recorded by ‘clumped isotope’ thermometry. Geochim Cosmochim Acta. 2008;72: 5351–5360. 10.1016/j.gca.2008.06.031

[65] Bar-Matthews M, Ayalon A, Vaks A, Frumkin A. Climate and Environment Reconstructions Based on Speleothems from the Levant In: Enzel Y, Bar-Yosef O, editors. Quaternary of the Levant: Environments, Climate Change, and Humans. Cambridge: Cambridge University Press; 2017 pp. 151–164.

[66] Ayalon A, Amit R, Enzel Y, Crouvi O, Harel M. Stable Carbon and Oxygen Isotope Signatures of Pedogenic Carbonates in Arid and Extremely Arid Environments in the Levant In: Enzel Y, Bar-Yosef O, editors. Quaternary of the Levant: Environments, Climate Change, and Humans. Cambridge: Cambridge University Press; 2017 pp. 423–432. Available: 10.1017/9781316106754.049

[67] Aloni R, Orshan G. Vegetation map of the lower Galilee. Isr J Bot. 1972;21: 209–227.

[68] Jaubert J, editor. Les Chasseurs d’Aurochs de La Borde: un site du Paléolithique moyen (Livernon, Lot). Paris: Editions de la Maison des sciences de l’homme; 1990.

[69] Pop E, Bakels C. Semi-open environmental conditions during phases of hominin occupation at the Eemian Interglacial basin site Neumark-Nord 2 and its wider environment. Quat Sci Rev. 2015;117: 72–81. 10.1016/j.quascirev.2015.03.020

[70] White M, Pettitt P, Schreve D. Shoot first, ask questions later: Interpretative narratives of Neanderthal hunting. Quat Sci Rev. 2016;140: 1–20. 10.1016/j.quascirev.2016.03.004

[71] Van Vuure CT. History, morphology and ecology of the Aurochs (Bos taurus primigenius). Lutra. 2002;45: 1–45.

[72] Shirli Bar-David, David Saltz, Tamar Dayan. Predicting the spatial dynamics of a reintroduced population: the persian fallow deer. Ecol Appl. 2005;15: 1833–1846. 10.1890/04-0798

[73] Mendelssohn H, Yom-Tov Y. Mammalia of Israel. Israel Acedemy of Sciences and Humanities; 1999.

[74] Boëda E, Geneste JM, Meignen L. Identification de chaînes opératoires lithiques du Paléolithique ancien et moyen. Paléo. 1990;2: 43–79.

[75] Hovers E. The many faces of cores-on-flakes: a perspective from the Levantine Mousterian In: McPherron SP, editor. Cores or Tools? Alternative Approaches to Stone Tool Analysis. Newcastle, UK: Cambridge Scholars Press; 2007 pp. 42–74. 10.1016/j.jenvrad.2007.03.002

[76] Kuhn SL. Mousterian Lithic Technology. An Ecological Perspective. Princeton: Princeton University Press; 1995.

[77] Dubreuil L, Savage D. Ground stones: a synthesis of the use-wear approach. J Archaeol Sci. 2014;48: 139–153. 10.1016/j.jas.2013.06.023

[78] Vergès JM, Ollé A. Technical microwear and residues in identifying bipolar knapping on an anvil: experimental data. J Archaeol Sci. 2011;38: 1016–1025. 10.1016/j.jas.2010.11.016

[79] Goren-Inbar N, Sharon G, Alperson-Afil N, Herzlinger G. A new type of anvil in the Acheulian of Gesher Benot Ya‘aqov, Israel. Phil Trans R Soc B. 2015;370: 20140353 10.1098/rstb.2014.0353 26483531 PMC4614716

[80] Revedin A, Aranguren B, Becattini R, Longo L, Marconi E, Lippi MM, et al Thirty thousand-year-old evidence of plant food processing. Proc Natl Acad Sci. 2010;107: 18815–18819. 10.1073/pnas.1006993107 20956317 PMC2973873

[81] Oron M, Goren-Inbar N. Mousterian intra-site spatial patterning at Quneitra, Golan Heights. Quat Int. 2014;331: 186–202. 10.1016/j.quaint.2013.04.013

[82] Beaune de S. Pour une archéologie du geste Broyer, moudre, piler, des premiers chasseurs aux premiers agriculteurs, Paris. Paris: CNRS Éditions; 2000.

[83] Zaidner Y. Adaptive Flexibility of Oldowan Hominins: Secondary Use of Flakes at Bizat Ruhama, Israel. PLOS ONE. 2013;8: e66851 10.1371/journal.pone.0066851 23840539 PMC3689005

[84] Lev E, Kislev ME, Bar-Yosef O. Mousterian vegetal food in Kebara Cave, Mt. Carmel. J Archaeol Sci. 2005;32: 475–484. 10.1016/j.jas.2004.11.006

[85] Madella M, Jones MK, Goldberg P, Goren Y, Hovers E. The exploitation of plant resources by Neanderthals in Amud Cave (Israel): the evidence from phytolith studies. J Archaeol Sci. 2002;29: 703–719. 10.1006/jasc.2001.0743

[86] Hardy BL. Climatic variability and plant food distribution in Pleistocene Europe: Implications for Neanderthal diet and subsistence. Quat Sci Rev. 2010;29: 662–679. 10.1016/j.quascirev.2009.11.016

[87] Morin E, Speth JD, Lee-thorp J. Middle Palaeolithic Diets: A Critical Examination of the Evidence In: Lee-thorp J, Katzenberg MA, editors. The Oxford Handbook of the Archaeology of Diet. 2016 doi: 10.1093/ oxfordhb/9780199694013.013.24

[88] Rabinovich R, Hovers E. Faunal Analysis from Amud Cave: preliminary results and interpretations. Int J Osteoarchaeol. 2004;14: 287–306.

[89] Speth J. Middle Palaeolithic subsistence in the Near East. Farming. 2012;2012: 1–45. 10.3828/bfarm.2012.2.1

[90] Mentzer SM. Hearths and Combustion Features In: Gilbert AS, editor. Encyclopedia of Geoarchaeology. Springer Netherlands; 2017 10.1007/978-1-4020-4409-0_133

[91] Whelan RJ. The Ecology of Fire. Cambridge University Press; 1995.

[92] Frick JA, Hoyer CT, Floss F. Comparative heating experiments on flint from The Côte Chalonnaise, Burgundy, France. Anthropologie. 2012;50: 295–322.

[93] Patterson LW. Thermal damage of chert. Lithic Technol. 1995;20: 72–80.

[94] Hovers E. Territorial Behavior in the Middle Paleolithic of the Southern Levant In: Conard NJ, editor. Settlement Dynamics of the Middle Paleolithic and Middle Stone Age. Kerns Verlag Tübingen; 2001 pp. 123–152.

[95] Griggo C, Boëda E, Bonilauri S, Al-Sakhel H, Emery-Barbier A, Courty MA. A Mousterian Dromedary Hunting Camp: Level VI1aO at Umm el Tlel (El Kowm, Central Syria In: Bon Fr, Costamagno S, Valdeyron N, editors. Hunting Camps in Prehistory. University Toulouse II—Le Mirail P@lethnology, 3; 2011 pp. 103–129.

[96] Marks AE, Freidel DA. Prehistoric settlement patterns in the Avdat/Aqev area. Prehistory and paleoenvironments in the Central Negev, Israel. 1977.

[97] Speth JD, Meignen L, Bar-Yosef O, Goldberg P. Spatial organization of Middle Paleolithic occupation X in Kebara Cave (Israel): Concentrations of animal bones. Quat Int. 2012;247: 85–102. 10.1016/j.quaint.2011.03.001

[98] Yeshurun R, Marom N, Bar-Oz G. Differential Fragmentation of Different Ungulate Body-Size: A Comparison of Gazelle and Fallow Deer Bone Fragmentation in Levantine Prehistoric Assemblages. J Taphon. 2007;5: 137–148.

[99] Yeshurun R, Bar-Oz G, Kaufman D, Weinstein-Evron M. Purpose, Permanence, and Perception of 14,000-Year-Old Architecture: Contextual Taphonomy of Food Refuse. Curr Anthropol. 2014;55: 591–618. 10.1086/678275

[100] Lengyel G. Operational schemes in the upper Palaeolithic and Epipalaeolithic of Raqefet cave. Ann Oradea Univ Fasciola Hist-Archaeol Univ Din Oradea Fasc Istor-Arheol. 2009;19.

[101] Porat N, Jain M, Ronen A, Horwitz LK. A contribution to late Middle Paleolithic chronology of the Levant: New luminescence ages for the Atlit Railway Bridge site, Coastal Plain, Israel. Quat Int. 2017; 10.1016/j.quaint.2016.09.045

[102] Meignen L, Speth JD, Bar Yosef O. Stratégies de subsistance et fonction de site au Paléolithique moyen récent: apports de la séquence de Kébara (Mt Carmel, Israël). Paléorient. 2017;43: 9–47.

[103] Meignen L, Bar Yosef O. Les Outils lithiques moustériens de Kebara (fouilles 1982–1985) In: Bar Yosef O, Vandermeersch B, editors. Le Squelette Moustérien de Kebara 2. Paris: Cahiers de Paléoanthropologie CNRS; 1991 pp. 49–85.

[104] Meignen L, Bar Yosef O. Middle Paleolithic Variability in Kebara Cave, Israel In: Akazawa T, Aoki K, Kimura T, editors. The Evolution and Dispersal of Modern Humans in Asia. Tokyo: Hakusen-Sha; 1992 pp. 129–148.

[105] Hovers E. The lithic assemblages of Amud Cave: Implication for understanding the end of the Mousterian in the Levant In: Akazawa T, Aoki K, Bar-Yosef O, editors. Neandertals and Modern Humans in Western Asia. New York: Plenum Press; 1998 pp. 143–163.

[106] Sharon G. A Week in the Life of the Mousterian Hunter In: Nishiaki Y, Akazawa T, editors. The Middle and Upper Paleolithic Archeology of the Levant and Beyond. Singapore: Springer Singapore; 2018 pp. 35–47. 10.1007/978-981-10-6826-3_3

[107] Pagli M. Middle Palaeolithic variability in the Near East as a reflection of different settlement dynamics: A comparative study of Umm el Tlel, Yabroud I (Syria) and Ksar’Akil (Lebanon) In: Conard NJ, Delagnes A, editors. Settlement Dynamics of Middle Palaeolithic and Middle Stone Age, Volume IV. Tübingen: Kerns Verlag; 2015 pp. 145–170.

[108] Bar-Yosef O, Vandermeersch B, Arensburg B, Belfer-Cohen A, Goldberg P, Laville H, et al The excavations in Kebara Cave, Mt. Carmel. Curr Anthropol. 1992;33: 497–514.

[109] Druck D. Raw Material Exploitation (Flint) in the Cultures of the Carmel Nahal Mearot. MA thesis (Hebrew), Haifa University. 2004.

[110] Ekshtain R, Ilani S, Segal I, Hovers E. Local and Nonlocal Procurement of Raw Material in Amud Cave, Israel: The Complex Mobility of Late Middle Paleolithic Groups. Geoarchaeology. 2017;32: 189–214. 10.1002/gea.21585

[111] Ekshtain R. Reconstructing Middle Paleolithic Mobility in the Levant: A Raw Material Perspective. Ph.D. Dissertation, Hebrew University of Jerusalem. 2014.

[112] Ullman M, Hovers E, Goren Inbar N, Frumkin A. Levantine cave dwellers: geographic and environmental aspects of early humans use of caves: a case study from Wadi Amud, northern Israel In: Filippi M, Bosak P, editors. Proceeidings of 16th International Congress of Speleology Volume 1 Brno, Czech Republic; 2013 pp. 169–174.

[113] Bar-Yosef O, Meignen L. Kebara Cave, Mt Carmel, Israel: The Middle and Upper Paleolithic Archaeology, Part I [Internet] Meignen OB-Y& L, editor. American School of Prehistoric Research, Peabody Museum, Harvard University Press; 2007 Available: https://halshs.archives-ouvertes.fr/halshs-00184128

[114] Hovers E. Cultural ecology at the Neandertal site of Amud Cave, Israel In: Derevianko AP, Nokhrina TI, editors. Arkheologiya i paleoekologiav Evrasii [Archaeology and Paleoecology of Eurasia], a volume dedicated to V Ranov. Novosibirsk: Institute of Archaeology and Ethnography SB RAS Press; 2004 pp. 218–231.

[115] Meignen L, Bar-Yosef O, Speth JD, Stiner MC. Middle Paleolithic Settlement Patterns in the Levant In: Hovers E, Kuhn SL, editors. Transitions before the Transition. Springer US; 2006 pp. 149–169. Available: http://link.springer.com/chapter/10.1007/0-387-24661-4_9

[116] Boeda E, Geneste JM, Griggo C, Mercier N, Muhesen S, Peyss JL, et al A Levallois point imbedded in the vertebra of a wild ass (Equus africanus). Am Antiq. 1999;

[117] Hovers E. The exploitation of raw material at the Mousterian site of Quneitra In: Goren-Inbar N, editor. Quneitra: a Mousterian Site on the Golan Heights. Jerusalem: Institute of Archaeology; 1990 pp. 150–176.

[118] Zaidner Y, Centi LE, Prevost M, Shemer M, Varoner O. An Open-Air Site at Nesher Ramla, Israel, and New Insights into Levantine Middle Paleolithic Technology and Site Use In: Nishiaki Y, Akazawa T, editors. The Middle and Upper Paleolithic Archeology of the Levant and Beyond. Springer; 2018 pp. 11–33.

