# Supplementary material for: Persistent Neanderthal occupation of the open-air site of ‘Ein Qashish, Israel
Source: PLoS One. 2019 Jun 26;14(6):e0215668. doi: 10.1371/journal.pone.0215668 (PMC6594589; doi:10.1371/journal.pone.0215668)
Supplement: S1 section — (DOCX) [file pone.0215668.s001.docx]

# **S1 section: Site stratigraphy and sedimentology**

The stratigraphic sequence exposed at EQ in the 2013 excavation was dated by 36 optically stimulated luminescence (OSL) samples. The dates reported here include those initially published by ([1] SI 2) as well as additional new dates from Area A. All the dates were obtained in the Israel Geological Survey. Once preliminary ages were available, it became apparent that some samples did not conform to the stratigraphic order and repeated analysis was carried out. Dose rates for three of the four outlier samples were either much higher or much lower than the mean dose rate calculated from all samples (due possibly to high concentrations of clay or heavy minerals and to dilution with a low-dose-rate mineral, such as calcium carbonate (CaCO3), respectively). To obtain robust ages for individual units and a chronological framework for the site, ages from each unit across the sections were averaged, excluding the outliers (see Table 1 in the main text and S1 Table).

The stratigraphic scheme presented in this paper is based on correlations across all the excavation areas and trenches throughout the five years of fieldwork (Table 1 in main text). In the current paper we use the terminology of **‘**units**’** instead of **‘**layers**’** used in [2] and [1]. The field relations and stratigraphic integration among all excavations and trenches is presented in Fig 4 and Table 1 of the main text. Units are numbered (1-11) from the oldest to recent. The older part of the section – Unit 1 and Unit 2 –was exposed only in trench EQ-5 and in the key section in areas B and F. Designations of the stratigraphic units 2**–**5, which include the MP materials, are identical to [1]. Layer 6 of [1] is equivalent to Unit 11 in the present study. Unit 7 (of late Pleistocene age) and units 9-10 (of Holocene age) were observed *in situ* only in Area A of the 2013 excavation, whereas Unit 8 of late Pleistocene age was exposed in trenches EQ-1, EQ-2 and EQ-3 of the 2009-11 excavation and in Area A. In the 2013 excavation, the lateral exposure of the various stratigraphic units was relatively extensive, which allowed for sub-division of units 3 and 5, based on spatial and sedimentological differences.

## **Unit 1**

This alluvial unit is exposed only in trench EQ-5. The unit is >50 cm thick and is composed of black, compacted, matrix supported, pebbles and cobbles up to 15 cm in size. The lower boundary of this unit was not exposed. The clasts are composed of angular limestone, flint and chalk gravel. In places, the gravels are structured as small lenses associated with a yellowish sandy matrix. The unit, which contains MP artifacts and fauna was OSL dated to 71±4 ka

## **Unit 2**

This alluvial unit is 70 cm thick and exposed in trench EQ-5 and partly at the bottom of Area F of the 2013 excavation ([1]: SI 1 Fig 4). This unit is composed of black, massive, compacted clay that contains small occasional lenses of granules and small pebbles up to 2 cm in size, with yellowish sandy-clay matrix that contains Fe-Mn concretions. Its contact with Unit 1 is abrupt and wavy. The unit is devoid of lithic or faunal remains. It is OSL dated to 76±3 ka.

## **Unit 3**

Unit 3 was sub-divided into two subunits –3a and 3b - based on sediment color and presence of gravels ([1]: Fig 2).

### **Unit 3a**

The lower of the two sub-units, is between 40 and 130 cm thick. It is present at the 2009-2011 excavation, in trench TQ-1 (where it was at the bottom of the trench and therefore designated there as unit 1 ([3]: Fig 5; and see above), at the 2013 excavation and in trench EQ-5. Respectively, OSL ages are 66±4 ka, 63±3 ka, and 66±3 ka. In the 2013 excavation it was dated t0 62±3 ka, 70±3 ka and 74±4 ka. The unit is composed of black, heavy, massive, compacted, prismatic silty-clay with slickensides. It includes black Fe-Mn concretions, and is densely mottled with yellowish iron stains, indicating gley and a hydromorphic environment with reducing conditions. Some small gravels are dispersed in places in the form of small lenses as well as calcified rhizolites, gypsum crystals and occasional calcite veins along cracks. The unit contains flint artifacts and bones. Its lower contact with Unit 2 is clear and wavy. The chemical and mineralogical properties indicate that the origin of the sediments of this unit is probably the upper reaches of the Qishon basin [3,4].

Additional faunal finds from this unit include 27 remains of terrestrial mollusks from Area F.

### **Unit 3b**

This sub-unit, 30-80 cm thick, is present at the 2009-2011 excavation and in Trench TQ-1 (where it is designated as unit 2; [3]: Fig 5), and the 2013 excavation. It was OSL dated to 64±4 ka, and 53±3 ka, respectively. An OSL single grain age of 53±3 ka was also calculated for the exposure of this unit at the archaeological site, using the minimum age model. Based on the size and lithology of the gravel, the origin of the unit is related to the alluvial fan coming from Mt. Carmel directly into the archaeological locality. The younger age of the unit at the top of the archaeological site may also be related to younger particles from the overlying clay that percolated into and among the gravels of unit 2. In the 2013 excavation area, it was dated to 59±3 ka, 71±3 ka and 65±3 ka. This gravelly unit is composed of about 30% moderately-bedded, sub-rounded limestone, dolomite and chalk gravel, angular flint and weathered basalt gravel up to 10 cm in size. The black, massive, compacted silty-clay matrix contains yellowish iron stains in the form of mottling and Fe-Mn concentrations indicating hydromorphic, reducing conditions. Slickensides and cracks are filled with reddish brown clay (probably derived post-depositionally from the overlying Unit 4), and gypsum crystals, 0.5-1 cm in size. The contact with the underlying Unit 3a is clear owing to the presence of gravels. Based on the size and lithology of the gravel, the origin of the unit is related to the alluvial fan coming from Mt. Carmel directly into the archaeological locality [3], but the black clay matrix may also suggest another possible source of large floods from Nahal Qishon.

This unit also contains terrestrial mollusk shells (N=7).

## **Unit 4**

The unit, 30-130 cm thick, was exposed in trench EQ-5 and at the 2013 excavation and was OSL dated to about 66±3 ka and 45±2 ka. The Unit is inclined ~10° to the NE and clearly truncates Unit 3 in trench EQ-5 and in areas E1 and E2 of the 2013 excavation ([1]: SI 1 Fig 4), where it is underlain by a gravel layer. It is composed of reddish-brown, massive, compacted prismatic silty-clay with abundant rhizoliths. The clay mineralogy is illitic. It contains Fe-Mn oxide nodules, gley and Fe stains along slickensides, large gypsum crystals and some disorthic carbonate nodules.

The red color and the different clay mineralogy were related to extensive staining by oxidized iron combined with diagenetic transformation of the smectitic clay into illite [4,5]. The presence of rhizoliths and of gypsum crystals indicates that the sediments were previously exposed long enough to allow for plant growth and evaporation. Stahlschmidt et al. [4] suggest that Unit 4 represents a spatially-constrained (minimally ~30 x ~20 m) seasonal water body, at the edge of Wadi Qashish alluvial fan, over the floodplain of the Qishon with a reducing environment, evidenced by gley and hydromorphic features, which changed into an oxidized environment. The abrupt, erosional contact with Unit 3 indicates truncation prior to this water body. The unit is almost barren of archaeological remains.

## **Unit 5**

The unit was exposed in trenches TQ-1 and EQ-5 at the 2009-2011 excavation as a single unit 40 and 80 cm thick [3]. In the 2013 excavation, this unit was found in areas A and C (Fig 12 in the main text) where it was also separated into sub-units 5a and 5b based on color and carbonate content. The unit is composed of black silty-clay typical of Qishon sediments.

### **Unit 5a**

The unit is 70-100 cm thick and composed of grey, massive, cracked, compacted, prismatic silty-clay, inclined ~10° to NE. In trench TQ-1 it was dated to 64±4 ka, in trench EQ-5 to 65±3 ka; in the 2013 excavation sections of areas B-F and E-1 the dates are 67±4 ka, and 84±6 ka, respectively and in Area A - 62±3 ka. The unit is rich in calcite and contains Fe-Mn yellowish mottling, gley and some concretions, rhizoliths, and gypsum crystals. Disorthic carbonate nodules are present with an increase in frequency upwards. The boundary with the underlying Unit 4 is clear. Abundant flint items and animal bones were found, particularly at the interface with Unit 5b.

### **Unit 5b**

The unit is 50-110 cm thick, composed of grey-olive brown, cracked, massive, compacted and cemented silty-clay. It contains - 50-60% carbonate nodules and some carbonate bridges, which form a continuous, stage III+, well-developed carbonate horizon and indicates a dominance of calcite (see Table 1 in the main text and below). The nodules are disorthic, up to 3 cm in size and their frequency increase upward. Carbonate stains occur along slickensides and cracks. Some of the flint artifacts and bones are covered by carbonate coating. It was OSL dated to 54±5 ka at the 2013 excavation (Area F) and 54±2 ka in Area A. Unit 5b contains flint artifacts and animal bones. Micromorphological and isotopic analyses indicate that the carbonates were washed from the surface and percolated into the soil by pedogenic processes rather than by groundwater [4]. The boundary between Unit 5a and Unit 5b is gradual.

## **Unit 6**

This clay unit, 50-100 cm thick, was exposed in the 2009-2011 excavation, in trench TQ-1 (designated there as unit 4; [3]: Fig 5), and in area A of the 2013 excavations, and was OSL dated between 50±3 and 42±2 ka, 41±3 ka and 43±2 ka, respectively (Fig 4 in the main text). The unit is composed of black, heavy, massive, compacted clay. It contains about 20% carbonate nodules and gypsum crystals. The lower contact with unit 5b is abrupt and indicates an unconformity. In Area A, the contact is clear but not abrupt, probably because of the carbonate accumulation and pedogenesis. The unit contains rolled artifacts, the preservation of which suggests that they were probably transported over a short distance or heaved upsection by the expansion and contraction of the clay [6].

## **Unit 7**

This grayish-brown clay unit, about 70 cm thick, was exposed only in Area A and was OSL dated to 27±1 ka (Fig 4 in the main text). It is composed of prismatic, massive, compacted, very rich (40%) with large gypsum crystals up to 2 cm in size. It contains also some yellowish Fe stains and mottling, carbonate nodules and rhizolites. These properties indicate marsh/temporary evaporating water body. The contact with the underlying Unit 6 is gradual. No lithic or faunal material were uncovered from this unit.

## **Unit 8**

This reddish brown, alluvial complex of gravelly and clay layers, > 3m thick, was derived from Wadi Qashish alluvial fan [3]. It was documented in trenches TQ-1, TQ-2 and TQ-3 (units 5-6 of [3]: Fig 5), and was OSL dated to between 15.1±0.5 and 10.5±0.5 ka. In Area A, the unit is about 1 m thick, located at the bottom of a paleo-channel and dated to 12±1 ka (Fig 4 in the main text). The gravelly horizons (units 5 and 6b in trenches TQ-1 and TQ-2, [3]: Fig 5) are composed of 30-40% gravel, poorly bedded, subrounded to subangular limestone and dolomite pebbles and cobbles, angular flint clasts and occasional rounded basalt gravels up to 20 cm in size. The matrix is hydromorphic, dark-brown clay with gley and greyish-yellow mottling. The reddish-brown clay layers (units 6a and 6c, trenches TQ-1 and TQ-2, [3]: Fig 5) are massive and compacted. In trench TQ-1 ([3]: Fig 5) remnants of a stage I-II calcic horizon with dense carbonate nodules 1-2 cm in size overtops Unit 6, indicating a period of exposure and pedogenesis. The major component of Unit 8 is aeolian quartz, which constitutes between 45% and 53% of the bulk sediment. The dominant clay mineral is illitic IS typical of well-developed and leached Terra Rosa soils derived from the nearby Mt. Carmel. The contact with the underlying units is abrupt and indicates unconformity. The unit contains subangular flint artifacts transported from upstream.

## **Unit 9**

This dark brown, light alluvial clay unit, which contains gravels, is located within the paleochannel in Area A (Fig 4 in the main text), where it is about 80 cm thick and OSL dated to 9±0.4 ka. It was also observed in Area C in the 2013 excavation. The unit contains carbonate nodules and Chalcolithic pottery. The contact with the underlying units is erosional.

## **Unit 10**

This gravelly unit cuts through the underlying units. It is a part of a channel network that represents relatively large recent flow episodes in the Wadi Qashish alluvial fan, transporting pebbles and cobbles up to 15 cm in size. The unit, 60-80 cm thick, is exposed in trenches TQ-2 and TQ-3 (unit 8, [3]: Fig 5), in trench EQ-5 and in area A of the 2013 excavation, where it was OSL dated to 6.4±0.4 ka (Fig 4 in the main text). The contact with the underlying units is erosional. The date of this unit is better constrained by the presence of Roman-Byzantine pottery, which suggests an age younger than about 2000 years.

## **Unit 11**

This reddish-dark brown, compact, cracked, clay unit covers the entire site at all trenches and excavations. The unit contains a calcic grumusol soil, which has developed with time, and is partly disturbed by cultivation. It has prismatic- cuboid structure and slickensides and contains up to 10% gravel and large carbonate nodules. In places, where the accumulation of the sediments is continuous such as in area F in the 2013 excavation, the unit is up to 1.7 m thick and was dated to 9±1 ka. In contrast, in active areas such as seen in trenches TQ-2 and TQ-3 (unit 9, [3]: Fig 5), the accumulation of the sediments of this unit is disturbed, the unit is 0.5 - 1.2 m thick and chronologically spans <2000 years. In area A, the unit is 1 m thick and its age is <6.4±0.4 ka. The erosive contact with the underlying units is usually clear.

# **References**

1. Been E, Hovers E, Ekshtain R, Malinski-Buller A, Agha N, Barash A, et al. The first Neanderthal remains from an open-air Middle Palaeolithic site in the Levant. Sci Rep. 2017;7: 2958. doi:10.1038/s41598-017-03025-z

2. Barzilai O, Malinsky-Buller A, Ekshtain, R, Hovers E. `En Qhasish. Hadashot Arkheologiot. 2015;127. Available: 127 http://www.hadashot-esi.org.il

3. Greenbaum N, Ekshtain R, Malinsky-Buller A, Porat N, Hovers E. The stratigraphy and paleogeography of the Middle Paleolithic open-air site of ‘Ein Qashish, Northern Israel. Quat Int. 2014;331: 203–215. doi:10.1016/j.quaint.2013.10.037

4. Stahlschmidt MC, Nir N, Greenbaum N, Zilberman T, Barzilai O, Ekshtain R, et al. Geoarchaeological Investigation of Site Formation and Depositional Environments at the Middle Palaeolithic Open-Air Site of ‘Ein Qashish, Israel. J Paleolit Archaeol. 2018;1: 32–53. doi:10.1007/s41982-018-0005-y

5. Sandler A. Clay distribution over the landscape of Israel: From the hyper-arid to the Mediterranean climate regimes. CATENA. 2013;110: 119–132. doi:10.1016/j.catena.2013.05.016

6. Yaalon DH, Kalmar D. Dynamics of cracking and swelling clay soils: Displacement of skeletal grains, optimum depth of slickensides, and rate of intra-pedonic turbation. Earth Surf Process. 1978;3: 31–42. doi:10.1002/esp.3290030104
