# Supplementary material for: Persistent Neanderthal occupation of the open-air site of ‘Ein Qashish, Israel
Source: PLoS One. 2019 Jun 26;14(6):e0215668. doi: 10.1371/journal.pone.0215668 (PMC6594589; doi:10.1371/journal.pone.0215668)
Supplement: S2 section — (DOCX) [file pone.0215668.s002.docx]

# **S2 section: Calcic soils**

Calcic soils characterize arid, semi-arid and Mediterranean type climates. Over similar rock and location and in constant climate conditions, their development is a function of time. The accretion of carbonates in the soil profile represents the development of the calcic soil into a hard calcrete through time. Based on a set of morphological-pedological criteria, the process of accretion was divided into 4 stages [1–3]. Stages I-III represent the initial and progressive soil development, whereas stage IV represents the maximum development of a single phase soil, where a hard, thick and continuous B(K) horizon (calcrete) is formed. This horizon, which becomes plugged with carbonates with time, causes a dramatic reduction in infiltration, which limits the eluviation of carbonates down the soil profile. Additional, 2 stages, were added by Bachman and Machette [4] and Machette [5], which represent the most developed degrees of soil development of fracturing, breakdown of the calcrete and re-infilling by carbonates (stage V) and multi-depositional phases of carbonates and formation of thick indurated calcrete (Stage VI).

The definition of the stages follows a nomenclature/menu of the various parameters, which are described/defined in the field and partly in the lab [6].

The stages of development represent the time of soil exposure to climate conditions and therefore are important in defining unconformities. The time-scale of the stages is different in various climates and locations and therefore, involves dating in order to calibrate the stage of soil development.

# **References**

1. Gile LH, Peterson FF, Grossman RB. The K horizon; A master soil horizon of carbonate accumulation. Soil Sci. 1965;99: 74.

2. Gile LH, Peterson FF, Grossman RB. Morphological and genetic sequences of carbonate accumulation in desert soils. Soil Sci. 1966;101: 347.

3. Gile LH, Grossman RB. Morphology of the argillic horizon in desert soils of southern New Mexico. Soil Sci. 1968;106: 6.

4. Bachman GO, Machette MN. Calcic soils and calcretes in the southwestern United States [Internet]. U.S. Geological Survey; 1977. Report No.: 77–794. Available: http://pubs.er.usgs.gov/publication/ofr77794

5. Machette MN. Calcic Soils of the Southwestern United S. tates. In: Weide DL, Faber L, editors. Soils and Quaternary Geology of the Southwestern United States. Denver, CO: Geological Society of America; 1985. pp. 1–21.

6. Birkeland PW, Mchette MN, Holler KM. Soils as a tool for applied Quaternary Geology. Utah Department of Natural Resources: Utab Geological and Mineral Survey; 1991.

# 
