# Supplementary material for: Persistent Neanderthal occupation of the open-air site of ‘Ein Qashish, Israel
Source: PLoS One. 2019 Jun 26;14(6):e0215668. doi: 10.1371/journal.pone.0215668 (PMC6594589; doi:10.1371/journal.pone.0215668)
Supplement: S3 section — (DOCX) [file pone.0215668.s003.docx]

# **S3 section: Bone geochemistry**

In order to understand possible diagenetic changes affecting the faunal record, several geochemical methods were used (e.g., FTIR, sediment pH, and elemental analyses) [1,2]. Bone mineral crystallinity, represented by the values of the splitting factor, is a useful proxy of bone preservation [1]. The values of fresh bone are between 2.5 and 2.9, whereas higher values indicate post-depositional increase in bone crystallinity. At ‘Ein Qashish twenty-four bones that were analyzed in area B show that crystallinity values are similar across space and along the stratigraphic sequence, which suggests that bones from all areas of the site underwent similar processes of diagenesis [3] hence their spatial and stratigraphic distributions cannot be related to the effects of such processes.

Nir [4] conducted an experimental study that aimed to clarify whether the absence of burnt bones was due to diagenesis or lack of deposition. The experiment shows that burnt bones are more soluble than unburnt bones; that cancellous bones are more soluble than cortical bones; and that blackened cortical bones are more soluble than calcined cortical bone. The mechanism behind this differential solubility is related to the ratio between surface area and volume.

Bone heating followed by acid treatment experiment showed that any process completely dissolving burnt bones (especially calcined bones) would leave marks (e.g. rounded and incomplete bones, high splitting factor values in FTIR spectra, and total absence of cancellous bone parts) also on the unburnt bone. As we do not recognize any such marks on the EQ bones, we have no reason to assume diagenetic changes (related to acidity) are the cause of the lack of burnt bones.

# **References**

1. Weiner S, Bar-Yosef O. States of preservation of bones from prehistoric sites in the Near East: A survey. J Archaeol Sci. 1990;17: 187–196. doi:10.1016/0305-4403(90)90058-D

2. Asscher Y, Weiner S, Boaretto E. Variations in Atomic Disorder in Biogenic Carbonate Hydroxyapatite Using the Infrared Spectrum Grinding Curve Method. Adv Funct Mater. 2011;21: 3308–3313. doi:10.1002/adfm.201100266

3. Stahlschmidt MC, Nir N, Greenbaum N, Zilberman T, Barzilai O, Ekshtain R, et al. Geoarchaeological Investigation of Site Formation and Depositional Environments at the Middle Palaeolithic Open-Air Site of ‘Ein Qashish, Israel. J Paleolit Archaeol. 2018;1: 32–53. doi:10.1007/s41982-018-0005-y

4. Nir N. Bones and formation processes in the Middle Paleolithic open-air site of Ein Qashish. MA Thesis, The Hebrew University. 2016.
