# Supplementary material for: Persistent Neanderthal occupation of the open-air site of ‘Ein Qashish, Israel
Source: PLoS One. 2019 Jun 26;14(6):e0215668. doi: 10.1371/journal.pone.0215668 (PMC6594589; doi:10.1371/journal.pone.0215668)
Supplement: S1 Table — S2A Table. (DOCX) [file pone.0215668.s004.docx]

**S1 Table. OSL Laboratory data and ages for ‘Ein Qashish**

| ‏Lab  No. | Unit no. | Depth  (m) | K  (%) | U (ppm) | Th (ppm) | Ext. α (μGy/a) | Ext. β (μGy/a) | Ext. γ (μGy/a) | Cosmic  (μGy/a) | Total dose  (μGy/a) | Aliquots  used | Over-  dispersion | De  (Gy) | Age  (ka) |
| --- | --- | --- | --- | --- | --- | --- | --- | --- | --- | --- | --- | --- | --- | --- |
|  | **Excavation 2009-2011** | | |  |  |  |  |  |  |  |  |  |  |  |
| EQH-1 | 3a | 3.1 | 0.57 | 1.8 | 8.6 | 9 | 684 | 612 | 114 | 1449±62 | 18/18 | 15 | 96±4 | **66±4** |
| EQH-2  SG | 3b | 2.9 | 0.44 | 1.3 | 4.9 | 6 | 484 | 397 | 147 | 1034±43 | 19/19  302/400 | 25  47 | 119±7  55±2 | 115±9  **53±3** |
| EQH-3 | 6 | 2.65 | 0.79 | 1.9 | 9.5 | 10 | 837 | 699 | 151 | 1698±77 | 20/20 | 14 | 84±3 | **50±3** |
| EQH-4 | 6 | 2.4 | 0.78 | 1.8 | 9.3 | 10 | 860 | 714 | 156 | 1740±62 | 19/19 | 14 | 73±3 | **42±2** |
|  |  |  |  |  |  |  |  |  |  |  |  |  |  |  |
|  | **Trench TQ-1** | |  |  |  |  |  |  |  |  |  |  |  |  |
| EQH-5 | 3b | 3.75 | 0.76 | 2.1 | 8.0 | 10 | 812 | 654 | 133 | 1608±67 | 19/19 | 17 | 104±5 | **64±4** |
| EQH-6 | 5a | 3.1 | 0.71 | 2.0 | 8.0 | 10 | 773 | 635 | 144 | 1560±65 | 18/19 | 18 | 100±4 | **64±4** |
| EQH-7 | 6 | 2.4 | 0.79 | 1.9 | 9.3 | 11 | 877 | 725 | 156 | 1769±61 | 19/19 | 20 | 73±4 | **41±3** |
| EQH-8 | 8 | 1.8 | 0.83 | 2.1 | 8.3 | 11 | 902 | 712 | 168 | 1793±60 | 17/17 | 11 | 18.8±0.6 | **10.5±0.5** |
| EQH-9 | 3a | 4.5 | 0.66 | 2.7 | 10.3 | 12 | 861 | 778 | 122 | 1774±76 | 18/18 | 10 | 112±3 | **63±3** |
|  | **Trench TQ-2** | |  |  |  |  |  |  |  |  |  |  |  |  |
| EQH-10 | 8 | 4.0 | 1.08 | 2.0 | 9.1 | 10 | 1054 | 787 | 129 | 1981±69 | 16/17 | 10 | 29.8±0.9 | **15.1±0.7** |
| EQH-11 | 8 | 2.2 | 1.08 | 2.6 | 10.1 | 12 | 1141 | 886 | 160 | 2199±73 | 17/17 | 18 | 27.2±1.2 | **12.4±0.7** |

**Ein Qashish 2013 excavations**

| Lab  No. | Field sample no. | Unit no. | Depth  (m) | Water contents  (%) | Area / plot | K  (%) | U  (ppm) | Th  (ppm) | Ext. α  (μGy/a) | Ext. β  (μGy/a) | Ext. γ  (μGy/a) | Cosmic  (μGy/a) | Total dose  (μGy/a) | No.  aliquots | OD  (%) | De  (Gy) | CAM  Age  (Ka) | Comment | UWM  Age  (Ka) |
| --- | --- | --- | --- | --- | --- | --- | --- | --- | --- | --- | --- | --- | --- | --- | --- | --- | --- | --- | --- |
|  | **Trench EQ-5** | |  |  |  |  |  |  |  |  |  |  |  |  |  |  |  |  |  |
| EQH-12 | EQ-5 | 5a | 2.5 | 12.3 |  | 0.30 | 1.2 | 4.5 | 6 | 415 | 368 | 154 | 944±28 | 22/22 | 32 | 61±3 | **65±3** |  | 66±11 |
| EQH-13 | EQ-4 | 4 | 3.0 | 35.8 |  | 1.08 | 2.3 | 12.6 | 11 | 953 | 796 | 145 | 1905±52 | 20/20 | 17 | 126±6 | **66±3** |  | 68±14 |
| EQH-14  SG | EQ-3 | 3a | 4.0 | 26.9 |  | 0.55 | 2.1 | 9.3 | 10 | 671 | 622 | 129 | 1433±38 | 19/19  59/140 | 17  34 | 104±4  94±3 | 72±4  **66±3** | 42% 2/4 comp | 73±14 |
| EQH-15 | EQ-2 | 2 | 4.5 | 26.6 |  | 0.55 | 2.3 | 8.8 | 10 | 682 | 621 | 122 | 1435±41 | 20/20 | 8 | 110±3 | **76±3** |  | 77±9 |
| EQH-16  SG | EQ-1 | 1 | 5.4 | 28.1 |  | 0.60 | 3.2 | 9.4 | 12 | 801 | 724 | 111 | 1648±49 | 21/23  58/138 | 34  40 | 112±5  117±5 | 68±4  **71±4** | 42% 3/4 comp | 70±16 |

| **Main Excavations 2013** | | | | |  |  |  |  |  |  |  |  |  |  |  |  |  |  |  |
| --- | --- | --- | --- | --- | --- | --- | --- | --- | --- | --- | --- | --- | --- | --- | --- | --- | --- | --- | --- |
| EQH-40 | QY-3a | 3a | 4.5 | 27.2** | F | 0.62 | 2.3 | 9.9 | 10 | 730 | 668 | 122 | 1531±49 | 17/17 | 10 | 95±3 | **62±3** |  | 62±7 |
| EQH-41 | QY-3b | 3b | 3.9 | 25.3** | F | 0.65 | 2.7 | 11.0 | 12 | 820 | 761 | 131 | 1724±56 | 17/17 | 14 | 102±4 | **59±3** |  | 59±9 |
| EQH-42 | QY-4 | 4 | 3.6 | 25** | F | 1.16 | 2.6 | 14.0 | 13 | 1144 | 962 | 135 | 2255±55 | 16/17 | 11 | 101±3 | **45±2** | **^+^65** | 45±5 |
| EQH-43 | QY-5a | 5a | 2.4 | 24.7** | F | 0.60 | 2.0 | 10.1 | 10 | 713 | 662 | 156 | 1540±39 | 17/17 | 18 | 103±5 | **67±4** |  | 68±14 |
| EQH-44 | QY-5b | 5b | 1.9 | 24.6** | F | 0.35 | 1.35 | 5.6 | 6 | 429 | 392 | 166 | 993±23 | 16/17 | 26 | 83±5 | **83±5** | **^+^54** | 86±20 |
| EQH-45 | QY-6 | 11 | 1.4 | 15* | F | 0.56 | 1.6 | 7.9 | 9 | 675 | 592 | 176 | 1452±58 | 17/17 | 39 | 14±1 | **9±1** |  | 10.0±3.9 |
| EQH-46 | QY-15 | 4 | 3.4 | 25** | E1 | 0.65 | 0.7 | 3.8 | 4 | 492 | 325 | 139 | 959±25 | 15/17 | 40 | 105±5 | **109±6** | **^+^68**  Y=76 | 111±20 |
| EQH-47 | QY-16 | 5a | 2.3 | 24.6** | E1 | 0.62 | 1.9 | 8.9 | 9 | 692 | 614 | 158 | 1473±47 | 17/17 | 25 | 124±8 | **84±6** | **^+^80** | 87±23 |
| EQH-48 | QY-17 | 3a | 4.1 | 22.2** | B3 | 0.60 | 1.9 | 10.0 | 10 | 716 | 663 | 128 | 1516±54 | 17/17 | 11 | 113±4 | **74±4** |  | 75±11 |
| EQH-49 | QY-18 | 3b | 3.6 | 22.3** | B2 | 0.66 | 1.9 | 10.8 | 10 | 764 | 704 | 135 | 1614±41 | 17/17 | 15 | 105±4 | **65±3** |  | 66±11 |
| EQH-50  SG | QY-19 | 3b | 3.9 | 14.8** | B2 | 0.42 | 1.6 | 6.7 | 8 | 567 | 509 | 131 | 1209±26 | 17/17  2/3 57% | 13  30 | 116±4  85±2 | 96±4  **71±3** |  | 97±15 |
| EQH-51 | QY-20 | 3a | 4.2 | 26.9** | B2 | 0.59 | 2.3 | 11.6 | 11 | 753 | 731 | 127 | 1621±54 | 17/17 | 10 | 113±3 | **70±3** |  | 70±9 |
| EQH-52 | QY-14 | 4a | 3.8 | 26.3** | E1 | 1.25 | 2.3 | 11.5 | 11 | 1099 | 850 | 132 | 2029±63 | 17/17 | 11 | 151±5 | **72±3** |  | 73±11 |
| EQH-53 | QY-13 | 9 | 2.3 | 21.3** | A | 0.67 | 1.6 | 6.6 | 7 | 665 | 529 | 158 | 1356±40 | 17/17 | 9 | 12.2±0.3 | **9±0.4** |  | 9.0±0.9 |
| EQH-54 | QY-12 | 8 | 2.6 | 19.6** | A | 0.54 | 1.4 | 5.6 | 6 | 564 | 453 | 152 | 1175±35 | 16/17 | 34 | 14±1 | **12±1** |  | 12.3±2.5 |
| EQH-55 | QY-11 | 7 | 1.4 | 29.4** | A | 0.56 | 1.6 | 6.4 | 7 | 556 | 463 | 176 | 1202±35 | 15/17 | 27 | 32±1 | **27±1** |  | 27.2±4.2 |
| EQH-56 | QY-10 | 5b | 2.4 | 23.5** | A | 0.55 | 1.5 | 7.0 | 7 | 584 | 500 | 156 | 1247±40 | 15/16 | 15 | 67±2 | **54±2** |  | 54±5 |
| EQH-57 | QY-9 | 10 | 1.6 | 14.8** | A | 0.65 | 2.1 | 6.7 | 9 | 753 | 608 | 172 | 1542±44 | 15/16 | 24 | 9.8±0.6 | **6.4±0.4** |  | 6.6±1.6 |
| EQH-58 | QY-8 | 6 | 2.2 | 19.4** | A | 0.57 | 1.9 | 6.6 | 8 | 653 | 543 | 160 | 1364±41 | 15/16 | 15 | 58±2 | **43±2** |  | 43±6 |
| EQH-59 | QY-7 | 5a | 3.3 | 19.2** | A | 0.78 | 1.9 | 9.9 | 10 | 837 | 713 | 140 | 1700±53 | 17/18 | 18 | 105±4 | **62±3** |  | 63±11 |

Grain size: 75-125 or 88-128 μm. “Over-dispersion” is an indication of the scatter in the sample. Ages are in thousands of years. Errors on the concentrations of K, U and Th are 5%, 5% and 10% of the values, respectively. Uncertainties on burial depths are estimated at 5% of the depth. SG – single grain analysis. Quartz was purified by sieving to the selected grain size, dissolving carbonates by 8% HCl, removing heavy minerals and most feldspars by magnetic separation, and dissolving the remaining feldspars and etching the quartz with 40% HF (for 40 min), followed by soaking in 16% HCl overnight to dissolve any fluorides which may have precipitated. De was measured on 1-2 mm aliquots using a modified single aliquot regenerative (SAR) protocol. Dose recovery tests over a range of preheats showed that a recovery of 100% can be obtained using a preheat of 10s @ 260^o^C, a test dose of ~9.3 Gy and a test dose preheat of 5 s @ 240 ^o^C. All samples show good recycling ratios within 8% of 1.0 and negligible IR signals. Alpha, beta and gamma dose rates were calculated from the radioactive elements measured by ICP MS (U&Th) or ICP-AES (K). Cosmic dose rates were estimated from burial depths. Aliquots used – the number of aliquots used for the average De out of the aliquots measured. OD- Over dispersion. De averages and errors were calculated using the central age model. Single grain De averages and errors were calculated using largest component of the finite mixture model. Water contents: *estimated - 20±5% for deeper samples (>2.5 m), and 15±3% for shallower samples; **measured. **^+^** Re-calculated ages (Been et al. 2017).
