# Supplementary material for: Persistent Neanderthal occupation of the open-air site of ‘Ein Qashish, Israel
Source: PLoS One. 2019 Jun 26;14(6):e0215668. doi: 10.1371/journal.pone.0215668 (PMC6594589; doi:10.1371/journal.pone.0215668)
Supplement: S2 Table — (a) The Lihtic assemblages of ‘Ein Qashsi according to stratigraphic units and excavation areas. (b) Technological breakdown of EQ assemblages by archaoelogical units. (DOCX) [file pone.0215668.s005.docx]

**S2a Table. The Lihtic assemblages of ‘Ein Qashsi according to stratigraphic units and excavation areas.**

| Debitage | | | | | | | Cores | | | | | | Tools | | | | | | Microartifacts (chips) | | | | | | TOTAL  (%) |
| --- | --- | --- | --- | --- | --- | --- | --- | --- | --- | --- | --- | --- | --- | --- | --- | --- | --- | --- | --- | --- | --- | --- | --- | --- | --- |
|  | A | B | C | E | F | All | A | B | C | E | F | All | A | B | C | E | F | All | A | B | C | E | F | All |  |
| Unit 5b | 1487  (36.6) |  | 557  (13.7) |  |  | 2044  (50.4) | 67  (1.6) |  | 16  (0.40) |  |  | 83  (2.0) | 63  (1.55) |  | 50  (1.2) |  |  | 113  (2.8) | 1101  (27.1) |  | 717  (17.7) |  |  | 1818  (44.8) | 4058  (100) |
| Unit 5a | 1236  (20) |  | 236  (5.5) | 910  (21.3) |  | 2382  (55.8) | 67  (1.6) |  | 11  (0.3) | 76  (1.8) |  | 154  (3.6) | 52  (1.2) |  | 6  (0.1) | 75  (1.8) |  | 133  (3.1) | 952  (22.3) |  | 150  (3.5) | 494  (11.16) |  | 1596  (37.4) | 4265  (100) |
| Unit 4 |  | 3  (5.9) |  | 28  (54.9) |  | 31  (60.8) |  |  |  |  |  |  |  |  |  | 2  (3.9) |  | 2  (3.9) |  |  |  | 18  (35.3) |  | 18 (35.3) | 51  (100) |
| Unit 3b |  | 716  (42.3) |  |  | 153  (9) | 869  (51.2) |  | 69  (4.1) |  |  | 18  (1.1) | 87  (5.2) |  | 54  (3.2) |  |  | 13  (0.8) | 67  (3.9) |  | 602  (35.5) |  |  | 68  (4) | 670  (39.5) | 1693  (100) |
| Unit 3a |  | 1223  (54) |  |  | 8  (0.3) | 1231  (54) |  | 85  (3.6) |  |  | 2  (0.1) | 87 |  | 74  (3.3) |  |  | 1  (0.1) | 75  (3.3) |  | 869  (38) |  |  |  | 869  (38) | 2262  (100) |
| TOTAL | 6557 | | | | | | 412 | | | | | | 340 | | | | | | 4971 | | | | | | **12,329** |

**S2b Table: Technological breakdown of EQ assemblages by archaoelogical units**

|  | **Unit 3a** | | **Unit 3b** | | **Unit 4** | | **Unit 5a** | | **Unit 5b** | |
| --- | --- | --- | --- | --- | --- | --- | --- | --- | --- | --- |
|  | N | % | N | % | N | % | N | % | N | % |
| **Cores** | 54 | 2.4 | 57 | 3.4 | 0 | 0.0 | 89 | 2.1 | 60 | 1.5 |
| **Cores on flakes** | 33 | 1.5 | 30 | 1.8 | 0 | 0.0 | 65 | 1.5 | 23 | 0.6 |
| **Cortical elements** | 116 | 5.1 | 102 | 6.0 | 2 | 3.9 | 234 | 5.5 | 210 | 5.2 |
| **Flakes** | 811 | 35.9 | 530 | 31.3 | 21 | 41.2 | 1586 | 37.2 | 1351 | 33.3 |
| **Levallois flakes** | 98 | 4.3 | 76 | 4.5 | 4 | 7.8 | 215 | 5.0 | 167 | 4.1 |
| **Maybe Levallois flakes** | 17 | 0.8 | 8 | 0.5 | 0 | 0.0 | 14 | 0.3 | 4 | 0.1 |
| **Blades** | 62 | 2.7 | 44 | 2.6 | 0 | 0.0 | 97 | 2.3 | 104 | 2.6 |
| **Levallois blades** | 28 | 1.2 | 28 | 1.7 | 0 | 0.0 | 27 | 0.6 | 23 | 0.6 |
| **Levallois points** | 17 | 0.8 | 14 | 0.8 | 0 | 0.0 | 15 | 0.4 | 12 | 0.3 |
| **Bladelets** | 7 | 0.3 | 8 | 0.5 | 1 | 2.0 | 27 | 0.6 | 36 | 0.9 |
| **CTE** | 64 | 2.8 | 49 | 2.9 | 3 | 5.9 | 144 | 3.4 | 120 | 3.0 |
| **Naturally backed knife** | 9 | 0.4 | 10 | 0.6 | 0 | 0.0 | 18 | 0.4 | 14 | 0.3 |
| **Varia** | 2 | 0.1 | 0 | 0.0 | 0 | 0.0 | 5 | 0.1 | 3 | 0.1 |
| **Tools** | 75 | 3.3 | 67 | 4.0 | 2 | 3.9 | 133 | 3.1 | 113 | 2.8 |
| **TOTAL** | 1393 | 61.6 | 1023 | 60.4 | 33 | 64.7 | 2669 | 62.6 | 2240 | 55.2 |
| **Chips** | 869 | 38.4 | 670 | 39.6 | 18 | 35.3 | 1596 | 37.4 | 1818 | 44.8 |
| **GRAND TOTAL** | 2262 | 100.0 | 1693 | 100.0 | 51 | 100.0 | 4265 | 100.0 | 4058 | 100.0 |
