# Supplementary material for: Persistent Neanderthal occupation of the open-air site of ‘Ein Qashish, Israel
Source: PLoS One. 2019 Jun 26;14(6):e0215668. doi: 10.1371/journal.pone.0215668 (PMC6594589; doi:10.1371/journal.pone.0215668)
Supplement: S3 Table — (DOCX) [file pone.0215668.s006.docx]

**S3 Table: Artifact densities in the archaeological units.**

| Layer | Volume | Debitage | Cores | Tools | Chips | Total |
| --- | --- | --- | --- | --- | --- | --- |
| 5b | 36.7 | 55.69 | 2.26 | 3.08 | 49.54 | 110.57 |
|  | 18.35* | 111.39 | 4.52 | 16.6 | 99.07 | 221.14 |
| 5a | 70.8 | 33.65 | 2.18 | 1.88 | 22.55 | 60.25 |
|  | 49.5* | 48.14 | 3.11 | 2.69 | 32.26 | 86.20 |
| 4 | 3.99 | 7.77 | 0.00 | 0.54 | 4.51 | 12.78 |
| 3b | 29.12 | 29.84 | 2.99 | 2.30 | 23.01 | 58.14 |
| 3a | 10.7 | 115.05 | 8.13 | 7.01 | 81.21 | 211.40 |

*Estimated layer volume accounting for postdeposition processes (see details in text)
